# Development and Validation of a Targeted Next-Generation Sequencing Gene Panel for Children With Neuroinflammation

**DOI:** 10.1001/jamanetworkopen.2019.14274

**Published:** 2019-10-30

**Authors:** Dara McCreary, Ebun Omoyinmi, Ying Hong, Ciara Mulhern, Charalampia Papadopoulou, Marina Casimir, Yael Hacohen, Rodney Nyanhete, Helena Ahlfors, Thomas Cullup, Ming Lim, Kimberly Gilmour, Kshitij Mankad, Evangeline Wassmer, Stefan Berg, Cheryl Hemingway, Paul Brogan, Despina Eleftheriou

**Affiliations:** 1 Infection, Inflammation and Rheumatology Section, University College London Great Ormond Street Institute of Child Health, London, United Kingdom; 2 Paediatric Neurology Department, Children NHS Foundation Trust, University College London Great Ormond Street Institute of Child Health, London, United Kingdom; 3 North East Thames Regional Genetics Laboratory, Great Ormond Street Hospital NHS Foundation Trust, London, United Kingdom; 4 Children’s Neurosciences Unit, Evelina London Children’s Hospital, Women’s and Children’s Department, Faculty of Life Sciences and Medicine, King’s College London, London, United Kingdom; 5 Immunology Department, Great Ormond Street Hospital NHS Foundations Trust, London, United Kingdom; 6 Paediatric Neuroradiology Department, Great Ormond Street Hospital for Children NHS Foundation Trust, London, United Kingdom; 7 Paediatric Neurology Department, Birmingham Children’s Hospital, Birmingham, United Kingdom; 8 Paediatric Rheumatology Department, University of Gothenburg, Gothenburg, Sweden; 9 Arthritis Research UK Centre for Adolescent Rheumatology, University College London, London, United Kingdom

## Abstract

**Importance:**

Neuroinflammatory disorders are a range of severe neurological disorders causing brain and spinal inflammation and are now increasingly recognized in the pediatric population. They are often characterized by marked genotypic and phenotypic heterogeneity, complicating diagnostic work in clinical practice and molecular diagnosis.

**Objective:**

To develop and evaluate a next-generation sequencing panel targeting genes causing neuroinflammation or mimicking neuroinflammation.

**Design, Setting, and Participants:**

Cohort study in which a total of 257 genes associated with monogenic neuroinflammation and/or cerebral vasculopathy, including monogenic noninflammatory diseases mimicking these entities, were selected. A customized enrichment capture array, the neuroinflammation gene panel (NIP), was created. Targeted high-coverage sequencing was applied to DNA samples taken from eligible patients referred to Great Ormond Street Hospital in London, United Kingdom, between January 1, 2017, and January 30, 2019, because of onset of disease early in life, family history, and/or complex neuroinflammatory phenotypes.

**Main Outcomes and Measures:**

The main outcome was the percentage of individuals with definitive molecular diagnoses, variant classification, and clinical phenotyping of patients with pathogenic variants identified using the NIP panel. The NIP panel was initially validated in 16 patients with known genetic diagnoses.

**Results:**

The NIP was both sensitive (95%) and specific (100%) for detection of known mutations, including gene deletions, copy number variants, small insertions and deletions, and somatic mosaicism with allele fraction as low as 3%. Prospective testing of 60 patients (30 [50%] male; median [range] age, 9.8 [0.8-20] years) presenting with heterogeneous neuroinflammatory phenotypes revealed at least 1 class 5 (clearly pathogenic) variant in 9 of 60 patients (15%); 18 of 60 patients (30%) had at least 1 class 4 (likely pathogenic) variant. Overall, a definitive molecular diagnosis was established in 12 of 60 patients (20%).

**Conclusions and Relevance:**

The NIP was associated with molecular diagnosis in this cohort and complemented routine laboratory and radiological workup of patients with neuroinflammation. Unexpected genotype-phenotype associations in patients with pathogenic variants deviating from the classic phenotype were identified. Obtaining an accurate molecular diagnosis in a timely fashion informed patient management, including successful targeted treatment in some instances and early institution of hematopoietic stem cell transplantation in others.

## Key Points

**Question:**

Is a customized gene panel associated with identification of monogenic disease-causing mutations in children with unclassified neuroinflammation?

**Findings:**

This cohort study developed a targeted sequencing approach using a panel of 257 genes, including those involved in neuroinflammation and mimics of neuroinflammation. In a cohort of 60 children with suspected genetic neuroinflammation, a molecular diagnosis was ascertained in 20% of patients, highlighting some unexpected genotype-phenotype associations and novel pathogenic variants.

**Meaning:**

Use of this gene panel may help obtain an accurate molecular diagnosis in a timely fashion to guide patient management, including early targeted treatment and early institution of allogeneic hematopoietic stem cell transplantation.

## Teaser

This cohort study describes the development and validation of a targeted next-generation sequencing gene panel to identify monogenic disease-causing mutations in children with unclassified neuroinflammation.

## Introduction

Neuroinflammatory diseases are increasingly recognized in the pediatric population and commonly present with a range of symptoms that include encephalopathy, seizures, and/or focal motor deficits.^1,2,3^ A monogenic cause for some neuroinflammatory conditions may be suspected, particularly if there is presentation early in life, consanguinity, and/or similar disease affecting other family members.^2,4^ Despite this, availability of routine genetic testing for monogenic neuroinflammation remains limited and expensive. Consequently, gene tests are usually requested individually and sequentially by clinicians, with definitive results acquired over months or years. Moreover, because there is considerable genotypic and phenotypic overlap for these diseases, particularly with neurometabolic and neurodegenerative disorders, there is often a diagnostic delay of several years, and some patients remain undiagnosed.^2^ Patients accrue significant irreversible central nervous system injury and may even die in this prediagnostic phase.^2^ Securing a definitive genetic diagnosis is thus important to enable timely therapeutic stratification of patients with monogenic neuroinflammation.

Next-generation genetic sequencing (NGS) targeted panels provide an opportunity to screen many genes known to cause neuroinflammation but have mainly been used in the context of research studies, with limited data on clinical outcomes for patients with neuroinflammation.^5,6,7,8,9^ We previously described^6^ a successful approach in developing a targeted NGS panel, the vasculitis and inflammation panel, to screen patients with autoinflammation and vasculitis. The objectives of this study were to design and validate an NGS targeted gene panel, the neuroinflammation panel (NIP), to screen patients with suspected genetic neuroinflammatory diseases or important genetic mimics of neuroinflammation, such as neurometabolic and neurodegenerative disorders, and to evaluate this approach as a routine diagnostic test for these conditions.

## Methods

### Study Population

This cohort study was approved by the National Research Ethics Service Committee London. All participants and parents provided written consent or assent as appropriate. We recruited patients referred nationally and internationally to a UK tertiary center for neuroinflammation (Great Ormond Street Hospital) between January 1, 2017, and January 30, 2019, presenting with a range of neurological features and neuroimaging and laboratory tests suggestive of neuroinflammatory disorders. Genetic sequencing was undertaken in the following instances: (1) in patients whose disease began early in life, (2) if there was a relevant family history (eg, of other affected individuals and/or consanguinity), and (3) in sporadic cases where there was clinical concern for genetic cause given the presenting phenotype (for instance, presence of intracerebral calcification, suggestive of a genetic interferonopathy). All participants had previously undergone extensive workup as part of their routine clinical care (eTable 1 in the Supplement).

For initial validation of the NIP, we recruited 16 patients with a known genetic neuroinflammatory disease to confirm the sensitivity for detection of these rare pathogenic variants. Genome in a Bottle, a DNA reference material, was also used to further evaluate the performance of the NIP, as described elsewhere.^10^

This study followed the Strengthening the Reporting of Observational Studies in Epidemiology (STROBE) reporting guideline for reporting observational studies.

### Targeted NIP Gene Panel and Capture Design

Genes were chosen following consideration of phenotypes referred to our specialized clinical service at Great Ormond Street Hospital and the published literature for genetic disorders of neuroinflammation. Important mimics of neuroinflammatory disorders and vasculitides, such as noninflammatory genetic vasculopathies (eg, caused by mutations in the elastin gene), neurodegenerative disorders, and neurometabolic disorders, were also included. Genes were considered in 9 broad disease subgroups (Table 1). The Agilent online SureDesign tool was used to design an NGS panel targeting 257 genes (eTable 2 in the Supplement includes the full gene list). The captured sequences included all coding and untranslated exons with at least 10 base pairs (bp) of the flanking intronic sequence to cover canonical splicing donor and acceptor sites. The designed probes are presented in eTable 3 in the Supplement.

**Table 1.** Summary of Disease Groups and Number of Genes in the Neuroinflammation Panel

Disease Group	No. of Genes
Arteriopathies	36
Autoinflammatory diseases	22
Complement disorders	16
Viral induced encephalomyelitis	1
Hemophagocytic lymphohistiocytosis	9
Primary immunodeficiencies	14
Monogenic Interferonopathies	21
Neuropathies	13
Inherited white matter and neurometabolic diseases	125
Total	257

### Targeted Gene Panel Sequencing

We extracted DNA from either EDTA blood or saliva using the Gentra Puregene extraction kit (Qiagen) and prepIT L2P kit (Genotek). Capture of targeted genes and regions with the Agilent QXT Target Enrichment system (Version C0) for Illumina sequencing was used. Briefly, genomic DNA was sheared by enzyme fragmentation and ligated with SureSelect Adaptor Oligo Mix. Fragment size was assessed using the TapeStation 2100 Bioanalyzer (Agilent). The adaptor ligated libraries were then amplified and hybridized to our customized SureSelect panel. Captured libraries were indexed, pooled, and sequenced as multiplex of 16 samples with the Illumina MiSeq sequencer in 150-bp paired-end mode. The Miseq reagent kit used was v3 (600 cycles). Sixteen samples were loaded per run.

**Figure 1.**
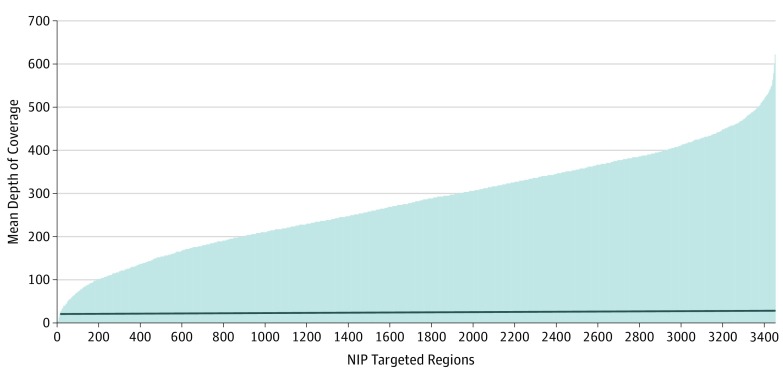
Depth of Coverage for Neuroinflammation Panel (NIP) Representative depth-of-coverage plot for all 76 (16 multiplexed run) captured samples. The captured regions are ordered according to mean depth of coverage. The horizontal line represents 30x level. Only 0.6% of the targeted regions had values less than 30x (mean [range] coverage, 280.02 [0-621.22]), including regions with no mapped reads.

Coverage from SureCall was used to access the read depths for all the captured regions per sample. The mean depth-of-coverage plot over the whole targeted regions for the 4 runs of 16 multiplexed samples showed that more than 99.4% of the captured regions had mean read depth greater than 30x, a commonly accepted cutoff for diagnostic purposes (Figure 1).

Additional baits were added to a total of 53 regions within 28 genes to improve coverage (eTable 4 in the Supplement). Although intrasample coverage showed some variation, coverage per region was highly reproducible between the different multiplexed runs (16 patient DNA samples per run).

A region was considered a low-coverage exon if any single nucleotide in the exon had a coverage less than 30x. Using that definition, 0.6% of the targeted regions, corresponding to 12 genes, had a mean depth of coverage less than 30x (mean [range] coverage, 280.02 [0-621.22]) (eTable 5 in the Supplement). Targeted regions for the *CORO1A, RANBP2, NCF1,* and *USP18* genes had reads that could not be confidently mapped to the genome (mapping quality score of 0) because of the pseudogene phenomenon.

### Bioinformatics Analysis

Read alignment, variant calling, and annotation were performed using SureCall version 4 software (Agilent) and an in-house pipeline, Genesis, developed at our North East Thames Regional Genetics laboratory. For both methods, the paired-end reads from the Illumina MiSeq instrument were mapped to the human genome (GRCh37) using Burrows-Wheeler Aligner–MEM software. See eMethods in the Supplement for details of Genesis, SureCall pipelines, and other immunological assays used.

The output variant call format file from SureCall was annotated using wANNOVAR, the web-based user-interfaced ANNOVAR tool from Wang Genomic Labs, which provided allele frequencies from public databases and in silico predictions of pathogenicity. Identified variants were evaluated for coverage using the Integrative Genomics Viewer (Broad Institute).

### Validation of NIP Capture Design Using DNA From Patients With Known Pathogenic Mutations

To evaluate the sensitivity and specificity of the NIP, we tested 16 anonymized positive control samples (8 [50%] male; median [range] age, 7.5 [1-16] years) with 19 known pathogenic mutations in 16 different genes previously identified using Sanger sequencing (Table 2). This sample was then run again, and the known variant was then detected. The Genome in a Bottle sample was also analyzed 3 times on 2 separate panel runs and confirmed adequate capture (data not shown).

**Table 2.** Summary of the Mutations Identified in 16 Positive Control Samples With Known Genetic Neuroinflammatory Diseases

Patient No.	Diagnosis	Known Gene Mutated	Nucleotide Change	Amino Acid Change	Zygosity	Read Depth	Allele Frequency		
							1000G	ESP650	ExAc
1	Glutaric acidemia IIC	*ETFDH* (NM_001281737)	c.578A>C	p.Glu193Ala	Homozygous	98	ND	ND	ND
2	Fabry disease	*GLA* (NM_000169)	c.274G>C	p.Asp92His	Heterozygous	230	ND	ND	ND
3	C1q deficiency	*C1QB* (NM_000491)	c.285del	p.Met95fs	Homozygous	355del	ND	ND	2.53 × 10^−5^
4	Krabbe disease	*GALC* (NM_000153)	30kb common del (Ex.11-17)	NA	Heterozygous	NA	ND	ND	ND
5	White matter disease	*ERCC6* (NM_000124)	c.1665dup	p.Thr556Aspfs*9	Heterozygous	218	ND	ND	ND
6	Metachromatic leukodystrophy	*ARSA* (NM_000487)	c.412del	p.His140fs	Heterozygous	568	ND	ND	ND
			c.917C>T	p.Thr306Met	Heterozygous	499	ND	1.8 × 10^−5^	1.01 × 10^−5^
7	Aicardi-Goutières syndrome	*TREX1* (NM_016381)	c.45C>G	p.Ile15Met	Homozygous	470	ND	ND	ND
8	Cockayne syndrome, type A	*ERCC8* (NM_000082)	c.844-2A>G	NA	Heterozygous	43	ND	ND	ND
9	Autoimmune lymphoproliferative disease	*FAS* (NM_000043)	c.539-2A>C	NA	Heterozygous	30	ND	ND	ND
10	Familial hemophagocytic lymphohistiocytosis	*STXBP2* (NM_006949)	c.1247-1G>C	NA	Homozygous	155	ND	0.0003	0.0002
11	Aicardi-Goutières syndrome	*TREX1* (NM_016381)	c.859_876del	p.287_292del	Homozygous	274	ND	ND	6.63 × 10^−5^
12	Cryopyrin associated periodic fever syndrome	*NLRP3* (NM_004895)	c.1699G>A	p.Glu567Lys (3% Mosaic)	Heterozygous	387	ND	ND	ND
13	X-linked lymphoproliferative disease	*SH2D1A* (NM_002351)	Gene del	NA	ND	ND	ND	ND	ND
14	Familial hemophagocytic lymphohistiocytosis	*STX11* (NM_003764)	Gene del	NA	ND	ND	ND	ND	ND
15	Deficiency of adenosine deaminase type 2	*ADA2* (NM_001282225)	c.752C>T	p.Pro251Leu	Heterozygous	151	2 × 10^−4^	0.0001	0.00003
			5′UTR -12233delC	5′UTR	Heterozygous	ND	0.07	ND	ND
16	Haploinsufficiency A20	*TNFAIP3* (NM_001270507)	c.811C>T	p.Arg271Ter	Heterozygous	34	ND	ND	ND

Abbreviations: *ADA2*, adenosine deaminase type 2; *ARSA*, arylsulfatase A; *C1QB*, complement C1 q B chain; del, deletion; *ERCC6*, excision repair 6, chromatin remodeling factor; *ERCC8*, excision repair 8, CSA ubiquitin ligase complex subunit; *ETFDH*, electron transfer flavoprotein dehydrogenase; *FAS*, fas cell surface death receptor; *GALC*, galactosylceramidase; *GLA*, galactosidase A; kb, kilobases; NA, not applicable; ND, no data; *NLRP3*, NLR family pyrin domain containing 3; *SH2D1A*, SH2 domain containing 1A; *STXBP2*, syntaxin binding protein 2; *STX11*, syntaxin 11; *TNFAIP3*, tumor necrosis factor α-induced protein 3; *TREX1*, 3 prime repair exonuclease 1.

### Pathogenicity Assessment of Identified Variants

First, we filtered out synonymous variants and then excluded common polymorphic variants with minor allele frequency of 1% or greater. Exceptions to this were 4 relatively common pathogenic variants: the perforin-1 (*PRF1*) monoallelic (NM_005041: p.Ala91Val) variant with minor allele frequency of 2% (but as high as 9% in other populations; this variant is known to impair cytotoxic function of natural killer cells); the NM_001065: p.Arg92Gln substitution in tumor necrosis factor–receptor superfamily member 1A (*TNFRSF1A*) present at 2% to 10% depending on ethnic background, but considered disease causing in some patients; the Mediterranean fever gene (*MEFV*; NM_000243: p.Glu148Gln) variant present at 0.13% depending on ethnic background; and the low-penetrant NM_004895:p.Val198Met variant in the nucleotide-binding domain and leucine-rich repeat containing family pyrin domain containing 3 (*NLRP3*) gene.

The public databases used to search for frequency of variants included the 1000 Genome Project, Exome Variant Server, and Exome Aggregation Consortium database. We investigated whether variants had been reported previously as pathogenic, their frequency in the population, segregation within the family (where samples were available), and predicted functional impact using SIFT, PolyPhen-2, Mutation taster, and Alamut-Batch version 2.11.

The identified variants were individually assessed and classified into pathogenicity groups (class 1, clearly not pathogenic; class 2, unlikely to be pathogenic; class 3, unknown significance; class 4, likely to be pathogenic; and class 5, clearly pathogenic) according to the Association for Clinical Genetic Science 2013 practice guidelines.^11^ Clinically actionable pathogenic variants were confirmed by Sanger sequencing where indicated and referred to our accredited genetic testing laboratory for validation. Familial segregation analysis for potentially pathogenic mutations was performed when possible. We returned to the referring clinicians’ standardized structured reports that summarized the classification of all identified rare variants (not only class 4 or 5, but also variants of unknown significance) and included any relevant published references.

### Statistical Analysis

Continuous variables are summarized as median and range. Categorical variables are presented as percentages and frequencies. Sensitivity and specificity were calculated using SPSS statistical software version 21 (IBM).

## Results

### Performance of NIP in Patients With Known Diagnosis

The initial NIP run was able to blindly identify 18 of the 19 (sensitivity of 95% and specificity of 100%) known pathogenic mutations in the 16 control patient samples, including an *NLRP3* (NM_004895: p.Glu567Lys) somatic mosaic mutation with allelic fraction of 3%. We were also able to identify copy number variation in the galactosylceramidase (*GALC* [NM_000153]) gene that had a 30-kilobase (kb) common deletion between exons 11 and 17 in heterozygous state. There was 1 of 19 variants (5.26%) that was not detected in this initial blinded analysis: the adenosine deaminase type2 *(ADA2)* gene UTR (NM_001282227: c.-12233delC [5′UTR]). We subsequently modified the capture design in NIP to include this region (*ADA2* 5′UTR variants:[NM_001282227: c.-12233delC] is at position: Chr 22:17702674).

### Performance of NIP in Patients With Unknown Diagnoses

Between January 1, 2017, and January 30, 2019, a total of 135 patients (95 [70%] male; median [range] age, 9.2 [0.6-20] years) were referred to the neuroinflammation service at Great Ormond Street Hospital. Neuroinflammation gene panel testing was undertaken on 60 patients (30 [50%] male; median [range] age, 9.8 [0.8-20] years) with suspected genetic neuroinflammation. Detailed descriptions of these patients are provided in eTable 6 and eTable 7 in the Supplement. We identified a total of 706 rare variants (median [range], 11.7 [4-27] variants per patient).

### Pathogenic Variants (Class 4 and Class 5)

Nine of 60 patients (15%) had at least 1 clearly pathogenic (class 5) variant and 18 of 60 patients (30%) had at least 1 likely pathogenic (class 4) variant (eTable 6 in the Supplement). A definitive molecular diagnosis was ascertained in 12 of 60 patients (20%). Variants identified in these 12 patients fulfilled the pathogenicity criteria from literature evidence, supported by confirmatory functional laboratory data supporting disease-genotype concordance, as described here and in eTable 6 in the Supplement.

Patient 21 (eTable 6 in the Supplement) was of nonconsanguineous descent and presented with fevers, refractory seizures, and 4-limb motor dystonic disorder following an episode of infectious gastroenteritis. Serial brain magnetic resonance imaging (MRI) revealed marked cerebral atrophy and signal change in basal ganglia, followed by cystic degeneration of the basal ganglia, internal capsule, and midbrain on subsequent scans. The patient was found to have a heterozygous mutation in the Fas cell surface death receptor (*FAS*) gene (NM_003824:c.517G>A; [p.Glu173Lys]), previously described in patients with autoimmune lymphoproliferative syndrome.^12,13^ This patient is currently undergoing assessment for allogeneic hematopoietic stem cell transplantation (HSCT).

**Figure 2.**
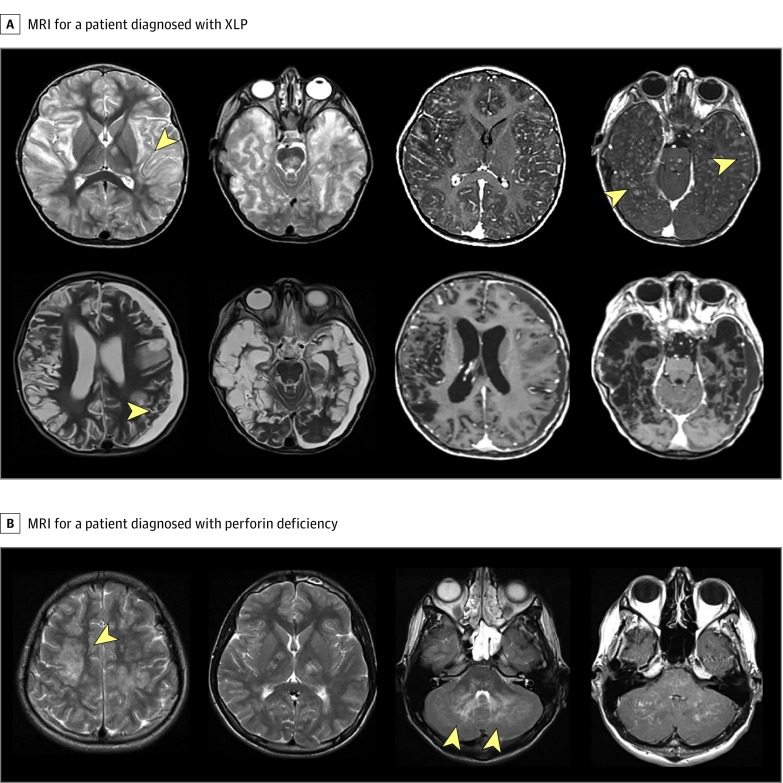
Neuroimaging Studies for 2 Patients Diagnosed With Primary Hemophagocytic Lymphohistiocytosis Following Sequencing on Neuroinflammation Gene Panel A, Magnetic resonance imaging (MRI) axial T2-weighted images from a patient diagnosed with X-linked lymphoproliferative (XLP) syndrome type 1 due to heterozygous c.163C>T variant in the SH2 domain containing 1A (SH2D1A) gene. In the top row, the 2 images to the left show extensive involvement of the subcortical white matter (arrowhead) affecting both cerebral hemispheres, thalami, and brainstem. To the right, 2 postgadolinium contrast images show extensive punctate enhancement (arrowheads) in the subcortical areas. In the bottom row, the 2 images on the left show follow-up imaging 7 months after initial presentation with marked brain atrophy with leukomalacia and a left-sided subdural collection (arrowhead). B, Brain MRI for a patient with perforin deficiency due to c.731T>G and c.694C>T variants in perforin gene. Signal changes are noted in both cerebral hemispheres, also affecting the capsular regions. Arrowheads indicate cerebellar signal abnormality.

Patient 26 (eTable 6 in the Supplement) was of nonconsanguineous descent and presented with severe encephalopathy, recurrent seizures, aphasia, and neutropenia (neutrophil count, 800/μL; reference range, 1500-8500/μL [to convert to ×10^9^ per liter, multiply by 0.001]). Serial MRI of the brain revealed progressive widespread bilateral white matter disease with significant brain volume loss (Figure 2A). The patient received corticosteroids (intravenous methylprednisolone, 30 mg/kg/d for 3 days, followed by oral prednisolone, 2 mg/kg/d) and intravenous cyclophosphamide (500-750 mg/m^2^ for 3-4 doses) but had poor clinical response and MRI progression of white matter disease. Neuroinflammation gene panel testing identified a heterozygous variant in the SH2 domain containing 1A (*SH2D1A*) gene (NM_002351: c.163C>T [p.Arg55.Ter]) previously described in association with X-linked lymphoproliferative disease type 1.^14,15^ Flow cytometric analysis of signaling lymphocytic activation molecule–associated protein (SAP) showed absent lymphocyte SAP expression, confirming the diagnosis of X-linked lymphoproliferative disease type 1 (eFigure, A in the Supplement). The patient underwent successful allogeneic HSCT. The child’s sibling (eFigure, B in the Supplement) also had the same mutation and has been referred for consideration of preemptive HSCT.

Patient 28 (eTable 6 in the Supplement) was a nonconsanguineous child presenting with refractory seizures, fevers, and mild erythematous skin rashes. Brain MRI showed white matter disease; brain biopsy showed a chronic inflammatory process affecting both white and gray matter. The patient had persistently raised C-reactive protein (90 mg/L; reference range <10 mg/L [to convert to nanomoles per liter, multiply by 9.524]) and elevated serum amyloid A (210 mg/L; reference range <10 mg/L) and had been previously treated with corticosteroids (2 mg/kg/d; weaned off over 8 weeks) and mycophenolate mofetil (1200 mg/m^2^/d), with partial efficacy. Neuroinflammation gene panel testing revealed the variant in *NLRP3* (NM_004895:c.214G>A [p.Val72Met]), previously reported in association with Muckle-Wells syndrome.^16^ The patient’s medication was changed to IL-1 blockade (anakinra) with normalization of C-reactive protein and serum amyloid A and stable MRI brain imaging appearances.

Patients 22, 29, 40, and 42 (eTable 6 in the Supplement) all were considered to have a neuroinflammatory disease radiologically classified as chronic lymphocytic inflammation with pontine perivascular enhancement responsive to steroids (CLIPPERS) syndrome and were treated with corticosteroids and multiple immunosuppressive medications. Patients 22 and 29 were both identified to have homozygous variants in *PRF1* (NM_005041) associated with hemophagocytic lymphohistiocytosis (HLH). We confirmed reduced perforin expression in patient 29 (Figure 2B); perforin expression testing in patient 22 was not possible, as the results of NIP were available after the individual died. Patient 40 had 2 variants in *STXBP2* (NM_006949: c.1621G>A; p.Gly541Ser and c. 1247-1G>C) previously described in association with HLH^17^; defective CD107 degranulation was confirmed. Patient 42 had 2 variants (NM_004580: c.550C>T [p.Arg184Ter] and NM_004580: c.259G>C [p.Ala87Pro]) in member RAS oncogene family (*RAB27A*) suggestive of Griscelli syndrome.^18^ Hair shaft examination revealed abnormal pigmentation (eFigure, C in the Supplement) and CD107 degranulation assay was defective. In view of these findings, patients 29, 40, and 42 were all subsequently fast-tracked to allogeneic HSCT.

Patient 31 was a child of consanguineous descent with proptosis, retinal ischemia, external ophthalmoplegia, and retinal angiopathy. The patient had a heterozygous mutation in DNA polymerase gamma 2, accessory subunit (*POLG2)* (NM_007215 c.1105A>G, p.Arg369Gly), previously described in association with dominant progressive external ophthalmoplegia.^19,20^

Patient 32 was of nonconsanguineous descent and presented with an unclassified autoinflammatory disease with recurrent fever, elevated acute phase reactants, headache, rashes, frequent respiratory tract infections, and cerebrospinal fluid (CSF) lymphocytosis (>100/μL [to convert to ×10^9^ per liter, multiply by 0.001]). This patient had compound heterozygous variants in phospholipase c gamma 2 (*PLCG2*) (NM_002661 c.1444T>C; p.Try482His and c.1712A>G, p.Asn571Ser). Monoallelic variants in *PLCG2* are associated with an autoinflammatory condition referred to as autoinflammation, antibody deficiency, and immune dysregulation syndrome (APLAID).^6,21^ Cosegregation analysis is ongoing in family members. Treatment with IL-1 blockade is being considered at the time of writing.

Patient 33 was a child of consanguineous descent who presented with a squint, vision loss, and left-sided hemiplegia associated with bilateral symmetrical parieto-occipital cortical and subcortical lesions affecting both gray and white matter disease, with edema with hemorrhagic transformation on brain MRI. The patient had splicing variants in *FOLR1* (NM_016725: c.493 + 2 T > C) and low CSF tetrahydrofolate levels (25 nmol/L; reference range, 40-128 nmol/L) but normal serum folate levels compatible with cerebral folate receptor deficiency.^22^ This individual was given folinic acid treatment with neurological stability at 7 months’ follow-up and recovery of CSF tetrahydrofolate levels (85 nmol/L).

Patient 36 was a nonconsanguineous child with neonatal meningoencephalitis, recurrent seizures, CSF lymphocytosis (>100/μL), and elevated CSF neopterins (105 nmol/L; reference range <65 nmol/L). Brain MRI showed bilateral widespread white matter disease; screening for congenital infection was negative. The patient had 2 variants in the ribonuclease H2 subunits B (*RNASEH2B)* gene (NM_024570: c.179T>G, p.Leu60Arg; and c.529G>A, p.Ala177Thr) linked to Aicardi-Goutières syndrome type 2.^23,24^ Other family members are currently undergoing genetic screening, and treatment with oral Janus kinase 1 and 2 inhibition is currently being considered.^25,26^

Patient 41 was a nonconsanguineous child with severe developmental delay and neuroblastoma-associated opsoclonus myoclonus in infancy. This child presented with a lupus-like disorder characterized by cutaneous vasculitis, malar rash, raised inflammatory markers, and positive autoantibodies (ANA, 1:400; anti-dsDNA, 580 IU/mL [reference range <70 IU/mL]; anti-ribo-P antibody positive). This patient had a heterozygous variant in interferon-induced helicase C domain containing protein 1 (*IFIH1)* (NM_022168: c.2336G>A, [p.Arg779His]) previously described as associated with Aicardi-Goutières syndrome type 7^27^ and with a monogenic lupus-like disorder.^28^ Treatment with Janus kinase inhibition has been started.

A total of 14 of 60 patients (23%) were carriers of class 4 or class 5 variants likely incidental to the observed phenotype. No definitive molecular diagnosis could be established in any of these cases (eTable 6 in the Supplement).

### Variants of Unknown Significance (Class 3)

A total of 363 unique variants of unknown significance in 102 genes were found in 34 patients (56%). Details of each patient and the various class 3 variants are presented in eTable 7 in the Supplement.

## Discussion

There is a broad differential diagnosis for suspected neuroinflammation, including true monogenetic neuroinflammatory disorders requiring early intervention with immunosuppression.^4,29,30,31,32^ Equally important are neurometabolic mimics, such as mitochondrial disease.^33^ For patients with true monogenetic neuroinflammation, further therapeutic stratification is now possible in the wake of an increasingly broad pharmacological armamentarium that includes corticosteroids, conventional immunosuppressants, biologic agents,^1,34,35,36,37^ and the recently introduced Janus kinase inhibitors.^25,26,38^ Some very severe immunological diseases, such as HLH, may initially present with a neuroinflammatory phenotype and ultimately require allogeneic HSCT if death is to be prevented.^39,40^ We therefore investigated the diagnostic utility of a targeted NIP, acknowledging the genetic heterogeneity among the many differential diagnoses that must be considered. The NIP proved to be reliable in the validation stage and was associated with molecular diagnosis in a cohort of undiagnosed pediatric patients referred to our specialist service with suspected neuroinflammation.

### Strengths

Our study had several strengths. A diagnosis was reached in 20% of participants, comparable to results reported by others in similar studies,^41,42^ including previous reports of patients with other complex neurological diseases. Obtaining an accurate molecular diagnosis in a timely fashion informed patient management, including successful targeted treatment (patients 28, 32, 33, 36, and 41) (eTable 6 in the Supplement) and instances of early institution of allogeneic HSCT that likely saved lives (patients 21, 26, 29, 40, and 42) (eTable 6 in the Supplement). Although applied to a pediatric cohort, our approach might also be diagnostically useful in adults, as neuroinflammatory phenotypes can be equally challenging diagnostically, and first presentation of genetic neuroinflammatory disease in adulthood is increasingly recognized.^8,43,44^

This study expands the genotypic and phenotypic spectrum of several disorders. Patient 26 presented with a predominantly neurological disorder characterized by severe encephalopathy as the initial sole presentation of X-linked lymphoproliferative syndrome. In addition, 4 patients who were all considered to have an unclassified neuroinflammatory disease radiologically classified as CLIPPERS syndrome were all found (somewhat surprisingly) to have HLH. Although the neurological manifestations of HLH have long been recognized in the context of typical systemic and hematological features, isolated neurological disease as the initial presentation of HLH is also increasingly recognized.^39,40^ The molecular diagnoses made in these cases allowed us to fast-track these patients to allogeneic HSCT and avoid unnecessary exposure to untargeted cytotoxic therapies.

### Limitations

This study had limitations. Thirty-four of 60 patients screened did not have any obviously pathogenic mutations. The patients with no pathogenic variants identified could have a nonmonogenetic complex cause for their neuroinflammatory phenotype, or variants may have been missed because of less efficient capture of GC-rich regions. It is also plausible that some disease-causing genes were not included in our NIP or that the mutations associated with disease were intronic, or within intergenic regions not covered by our panel.

Whole-exome sequencing (WES) or whole-genome sequencing may provide greater ability to diagnose and will be an area of future study. Use of targeted NGS panels in routine clinical practice still has some benefits over the use of clinical-grade WES, such as (1) reduced costs given that WES remains costly, particularly when a child and both parents have sequencing performed (cost of £412 [506 US dollars] per sample for WES and approximately £1236 [1519 US dollars] for 3 samples compared with £397 [488 US dollars] per sample for targeted NGS panel run); (2) a shorter turnaround time of results given that WES still requires time-consuming manual inspection and expert analysis; and (3) increased depth of coverage of targeted sequences with targeted NGS panels that may allow identification of somatic mosaicism. Improved sequencing techniques and bioinformatics pipelines may, however, improve efficiency of WES and whole-genome sequencing for use in routine clinical practice over the next few years.^45^

Another major challenge relates to functional readouts to support genetic findings. Elucidating the significance of class 3 variants is often not possible in routine clinical care because pertinent functional laboratory readouts may not be available. In addition, much faster turnaround times may often be clinically desirable for some patients with catastrophic neuroinflammatory presentation of potentially treatable cause (such as HLH).^46^

## Conclusions

We have described the diagnostic utility of a NGS targeted gene panel for neuroinflammation and neuroinflammatory mimics and identified at least 1 clearly pathogenic (class 5) variant in 15% of patients and 1 likely pathogenic (class 4) variant in 30% of patients; a firm molecular diagnosis was established in 20% of patients studied. Unexpected genotype-phenotype associations in patients with pathogenic variants deviating from the classic phenotype included a predominantly neurological disorder as the initial sole presentation of X-linked lymphoproliferative disease; autoimmune lymphoproliferative syndrome; and CLIPPERS-like radiological phenotype for patients with isolated central nervous system HLH. Obtaining an accurate molecular diagnosis in a timely fashion informed patient management, including successful targeted treatment in some instances and early institution of allogeneic HSCT in others.

## Notes

Supplement. **eMethods.** Additional Information **eTable 1.** List of Laboratory Investigations Organized in Context of Routine Clinical Care for Patients With Suspected Neuroinflammation **eTable 2.** Detailed List of Genes Included in Neuroinflammation Panel (NIP) **eTable 3.** SureDesign Description of Probes for NIP **eTable 4.** Regions Below the Depth of Coverage Threshold of 30X **eTable 5.** List of Regions With More Baits Added to Improve Coverage **eTable 6.** Clinical Features and Class 4 and 5 Genetic Variants Identified on Neuroinflammation Gene Panel (NIP) in Patients With Suspected Genetic Neuroinflammatory Disorders and Unknown Molecular Diagnoses **eTable 7.** Clinical Features and Class 3 Genetic Variants Identified on Neuroinflammation Gene Panel (NIP) in 34 Patients With Suspected Genetic Neuroinflammatory Disorders and Unknown Molecular Diagnoses **eFigure.** Further Tests in Patients With Isolated Neuroinflammation
