# Supplementary material for: Development and Validation of a Targeted Next-Generation Sequencing Gene Panel for Children With Neuroinflammation
Source: JAMA Netw Open. 2019 Oct 30;2(10):e1914274. doi: 10.1001/jamanetworkopen.2019.14274 (PMC6824223; doi:10.1001/jamanetworkopen.2019.14274)
Supplement: Supplement. — eMethods. Additional Information eTable 1. List of Laboratory Investigations Organized in Context of Routine Clinical Care for Patients With Suspected Neuroinflammation eTable 2. Detailed List of Genes Included in Neuroinflammation Panel (NIP) eTable 3. SureDesign Description of Probes for NIP eTable 4. Regions Below the Depth of Coverage Threshold of 30X eTable 5. List of Regions With More Baits Added to Improve Coverage eTable 6. Clinical Features and Class 4 and 5 Genetic Variants Identified on Neuroinflammation Gene Panel (NIP) in Patients With Suspected Genetic Neuroinflammatory Disorders and Unknown Molecular Diagnoses eTable 7. Clinical Features and Class 3 Genetic Variants Identified on Neuroinflammation Gene Panel (NIP) in 34 Patients With Suspected Genetic Neuroinflammatory Disorders and Unknown Molecular Diagnoses eFigure. Further Tests in Patients With Isolated Neuroinflammation [file jamanetwopen-2-e1914274-s001.pdf]

Supplementary Online Content

McCreary D, Omoyinmi E, Hong Y, et al. Development and validation of a targeted next-generation sequencing gene panel for children with neuroinflammation. *JAMA Netw Open*. 2019;2(10):e1914274. doi:10.1001/jamanetworkopen.2019.14274

eMethods. Additional Information

eTable 1. List of Laboratory Investigations Organized in Context of Routine Clinical Care for Patients With Suspected Neuroinflammation

eTable 2. Detailed List of Genes Included in Neuroinflammation Panel (NIP)

eTable 3. SureDesign Description of Probes for NIP

eTable 4. Regions Below the Depth of Coverage Threshold of 30X

eTable 5. List of Regions With More Baits Added to Improve Coverage

eTable 6. Clinical Features and Class 4 and 5 Genetic Variants Identified on Neuroinflammation Gene Panel (NIP) in Patients With Suspected Genetic Neuroinflammatory Disorders and Unknown Molecular Diagnoses

eTable 7. Clinical Features and Class 3 Genetic Variants Identified on Neuroinflammation Gene Panel (NIP) in 34 Patients With Suspected Genetic Neuroinflammatory Disorders and Unknown Molecular Diagnoses

eFigure. Further Tests in Patients With Isolated Neuroinflammation

This supplementary material has been provided by the authors to give readers additional information about their work.

© 2019 McCreary D et al. *JAMA Network Open.*

eMethods. Additional Information

Granule release assay

The granule release assay was performed according to Wheeler et al. (2010). Peripheral blood mononuclear cells (PBMC) were stimulated with IL-2 overnight, and then incubated with FITC-conjugated anti-CD107a antibody alone, or with anti-CD3 antibody or PHA, for 2 h. Samples were analyzed by flow cytometry and gating on lymphocytes by forward/side scatter. CD107a expression was analyzed on cytotoxic T cells (CD8+ and CD3+) and on NK cells (CD56+ and CD3−), and the increase in percentage of CD107a+ cells between unstimulated and stimulated samples was calculated.

XIAP and SAP Protein expression.

Normal healthy control and whole blood was fixed and permeabilized using Intraprep/Intraperm (Beckman Coulter) according to manufacturer's protocol. An isotype IgG (BD Biosciences), anti-SAP (Abnova) or anti-XIAP (BD Biosciences) was added at to each tube, incubated 15 minutes room temperature, washed with PBS/0.1mM EDTA/1%BSA followed by staining with anti-CD3, anti-CD56 and anti-CD8 (BD Bioscience) for 10 minutes at room temperature. Samples were then washed, fixed (Cell Fix, BD Bioscience) and acquired on a FACsCalibur (BD Bioscience). Using Celle Quest software (BD Bioscience), sequential gating was used to identify the CD8 population and then histogram analyses with SAP and XIAP overlaying the isotype. Controls have >50% positivity for SAP and XIAP.

Bioinformatics parameters used for both SureCall and Genesis pipelines.

Agilent SureCall (Version 4.0.1.46) analysis parameters:

SNPPET caller in SureCall was used to identify single nucleotide polymorphisms (SNPs), multiple nucleotide polymorphisms (MNPs). The parameters that SureCall uses to identify SNPs, MNPs, and indels are:

- QC Metric applied: true
- Report Regions Having Read Depth Below: 20
- Enable Trimming: true
- Quality Threshold for Trimming: 5
- Minimum Read Length Fraction: 30
- Maximum Read Length Fraction: 100
- Aligner applied: true
- Illumina: BWA MEM
- Mismatch Penalty: 4.0
- Base Quality Encoding: Auto Detect
- Score Threshold for a Match: 1.0
- Maximum Number of Mismatches: 2
- Output Filter: Unique Best Hits
- Constraint for maximal gap length: 100
- Minimum matching seed length: 19
- Remove Duplicates: true
- Region Padding applied: true
- Region Padding: 100
- BAQ SNP Caller applied: false
- SNPPET SNP Caller applied: true
- Quality value threshold: 0.3
- Use base quality recalibration: false

© 2019 McCreary D et al. *JAMA Network Open.*

- Minimum mapping quality for read: 30
- Minimum quality for base: 30
- Report multiple Alleles at a locus: false
- Quality value threshold (low frequency): 100
- Minimum Allele Frequency: 0.03
- Minimum number of reads supporting variant allele: 10

Parameters for SureCall data analysis for Copy Number Variation.

- QC Metric applied: true
- Report Regions Having Read Depth Below: 20
- Enable Trimming: true
- Quality Threshold for Trimming: 5
- Minimum Read Length Fraction: 30
- Maximum Read Length Fraction: 100
- Aligner applied: true
- Illumina: BWA MEM
- Mismatch Penalty: 4.0
- Base Quality Encoding: Auto Detect
- Score Threshold for a Match: 1.0
- Maximum Number of Mismatches: 2
- Output Filter: Unique Best Hits
- Constraint for maximal gap length: 100
- Minimum matching seed length: 19
- Remove Duplicates: true
- Region Padding applied: true
- Region Padding: 100
- BAQ SNP Caller applied: false
- SNPPET SNP Caller applied: true
- Quality value threshold: 0.3
- Use base quality recalibration: false
- Minimum mapping quality for read: 30
- Minimum quality for base: 30
- Report multiple Alleles at a locus: false
- Quality value threshold (low frequency): 100
- Minimum Allele Frequency: 0.01
- Minimum number of reads supporting variant allele: 10
- Minimum number of read pairs per barcode: 2
- Minimum number of reads supporting Denovo variant: 10
- Denovo variant quality: 0
- Denovo variant genotype quality: 10
- SNP Filter: true
- Absolute difference in max base quality observed for variant and wild type reads: 20:20:20
- Mutation Impact: Default
- Version of Annotation: V3

Parameters for Genesis data analysis

Genesis is an in-house pipeline, developed at NE Thames Regional Genetics laboratory. In short, paired end reads were aligned to the human genome (GRCh37) using Burrows-Wheeler Aligner (BWA)-MEM version 0.7.15 [47] and the variant calling was performed using Freebayes (sensitive settings; version 1.1.0; https://github.com/ekg/freebayes) using region of interest of the targeted genes. VEP87 and Gemini v0.20 were used for variant annotation and filtering. Variants were further annotated using Alamut-batch (version 1.9; http://www.interactive-biosoftware.com/alamut-batch/) and variants with ExAC frequency > 2% were filtered out. CNVs were called using a bioconductor package ExomeDepth (version 1.0.7; [48]) excluding sex chromosomes. Coverage depth was calculated using chanjo (version 2.3.2; https://github.com/robinandeer/chanjo).

© 2019 McCreary D et al. *JAMA Network Open.*

eTable 1. List of laboratory investigations organized in context of routine clinical care for patients with suspected neuroinflammation. CSF=cerebrospinal fluid. ANA=antinuclear antibodies. ENA=extranuclear antibodies. ANCA=antineutrophil cytoplasmic antibodies. MOG=myelin oligodendrocyte antibodies.

Full blood count and blood film
Erythrocyte sedimentation rate
C-reactive protein
Urea creatinine and electrolytes
Liver function tests
CSF glucose and protein, microscopy and culture.
CSF/serum oligoclonal bands
CSF neopterins
Clotting screen
C3 and C4 levels, complement function
IgG/IgM/IgG
ANA/dsDNA/ENA
ANCA
MOG antibodies
Anti-aquaporin4 antibodies
Urine for albumin: creatinine ratio.
Thyroid function and thyroid antibodies
Plasma amino acids, lactate, ammonia
For patients with suspected vasculopathy plasma homocysteine and alpha galactosidase levels
Muscle biopsy for respiratory chain enzymes in case of suspected mitochondrial disease

© 2019 McCreary D et al. *JAMA Network Open.*

eTable 2. Detailed list of genes included in Neuroinflammation panel (NIP).

Gene	*HGNC Symbol*	**Gene Name**	**Disease Group**	**Chr**	**Transcript reference from HGNC**
ACTA2	*ACTA2*	Actin alpha 2	Arteriopathies	10	NM_001613
BMPR2	*BMPR2*	Bone morphogenetic protein receptor type 2	Arteriopathies	2	NM_001204
CBL	*CBL*	Cbl proto-oncogene	Arteriopathies	11	NM_005188
CBS	*CBS*	Cystathionine-beta-synthase	Arteriopathies	21	NM_000071
CECR1	*CECR1*	Cat eye syndrome chromosome region 1/Adenosine deaminase 2	Arteriopathies	22	NM_001282225
COL3A1	*COL3A1*	Collagen type III alpha 1 chain	Arteriopathies	2	NM_000090
COL4A1	*COL4A1*	Collagen type IV alpha 1 chain	Arteriopathies	13	NM_001303110
ELN	*ELN*	Elastin	Arteriopathies	7	NM_000501
FBN1	*FBN1*	Fibrillin1	Arteriopathies	15	NM_000138
FBN2	*FBN2*	Fibrillin 2	Arteriopathies	5	NM_001999
FOXE3	*FOXE3*	Forkhead box E3	Arteriopathies	1	NM_012186
FOXP3	*FOXP3*	Forkhead box P3	Arteriopathies	X	NM_014009
GUCY1A3	*GUCY1A3*	Guanylate cyclase 1 soluble subunit alpha 1	Arteriopathies	4	NM_000856
HTRA1	*HTRA1*	HtrA serine peptidase-1 gene	Arteriopathies	10	NM_002775
LOX	*LOX*	Lysyl oxidase	Arteriopathies	5	NM_001178102
MAT2A	*MAT2A*	Methionine adenosyltransferase 2A	Arteriopathies	2	NM_005911
MFAP5	*MFAP5*	Microfibril associated protein 5	Arteriopathies	12	NM_003480
MYH11	*MYH11*	Myosin heavy chain 11	Arteriopathies	16	NM_001040113
MYLK	*MYLK*	Myosin light chain kinase	Arteriopathies	3	NM_053025
NOTCH1	*NOTCH1*	Notch 1	Arteriopathies	9	NM_017617
NOTCH3	*NOTCH3*	Notch 3	Arteriopathies	19	NM_000435
PRKG1	*PRKG1*	Protein kinase cGMP-dependent 1	Arteriopathies	10	NM_001098512
RNF213	*RNF213*	Ring finger protein 213	Arteriopathies	17	NM_020914
SAMD3	*SAMD3*	Sterile alpha motif domain containing 3	Arteriopathies	6	NM_152552
SKI	*SKI*	SKI proto-oncogene	Arteriopathies	1	NM_003036

© 2019 McCreary D et al. *JAMA Network Open.*

SLC2A10	*SLC2A10*	Solute carrier family 2 member 10	Arteriopathies	20	NM_030777
SMAD2	*SMAD2*	SMAD family member 2	Arteriopathies	18	NM_005901
SMAD3	*SMAD3*	SMAD family member 3	Arteriopathies	15	NM_005902
SMAD4	*SMAD4*	SMAD family member 4	Arteriopathies	18	NM_005359
TGFB2	*TGFB2*	Transforming growth factor beta 2	Arteriopathies	1	NM_003238
TGFB3	*TGFB3*	Transforming growth factor beta 3	Arteriopathies	14	NM_003239
TGFBI	*TGFBI*	Transforming growth factor beta induced	Arteriopathies	5	NM_000358
TGFBR1	*TGFBR1*	Transforming growth factor beta receptor 1	Arteriopathies	5	NM_000358
TGFBR2	*TGFBR2*	transforming growth factor beta receptor 2	Arteriopathies	3	NM_001024847
USB1	*USB1*	U6 snRNA biogenesis phosphodiesterase 1	Arteriopathies	16	NM_024598
YY1AP1	-	YY1 associated protein 1	Arteriopathies	1	NM_139118
COPA	*COPA*	Coatomer protein complex subunit alpha	Autoinflammatory	1	NM_004371
CTLA4	*CTLA4*	Cytotoxic T-lymphocyte associated protein 4	Autoinflammatory	2	NM_005214
IL1RN	*IL1RN*	Interleukin 1 receptor antagonist	Autoinflammatory	2	NM_173841
LPIN2	*LPIN2*	LPIN2	Autoinflammatory	18	NM_014646
MEFV	*MEFV*	Mediterranean Fever	Autoinflammatory	16	NM_000243
MVK	*MVK*	Mevalonate Kinase	Autoinflammatory	16	NM_000243
NLRP12	*NLRP12*	NLR family pyrin domain containing 12	Autoinflammatory	19	NM_144687
NLRP3	*NLRP3*	NLR family pyrin domain containing 3	Autoinflammatory	1	NM_004895
NOD2	*NOD2*	Nucleotide binding oligomerization domain containing 2	Autoinflammatory	16	NM_022162
OTULIN	*OTULIN*	OTU deubiquitinase with linear linkage specificity	Autoinflammatory	5	NM_138348
PLCG2	*PLCG2*	Phospholipase C gamma 2	Autoinflammatory	16	NM_002661
PLOD1	*PLOD1*	Procollagen-lysine,2-oxoglutarate 5-dioxygenase 1	Autoinflammatory	1	NM_000302

© 2019 McCreary D et al. *JAMA Network Open.*

PSMA3	*PSMA3*	Proteasome subunit alpha 3	Autoinflammatory	14	NM_002788
PSTPIP1	*PSTPIP1*	Proline-serine-threonine phosphatase interacting protein 1	Autoinflammatory	15	NM_003978
PYCARD	*PYCARD*	PYD and CARD domain containing	Autoinflammatory	16	NM_013258
RBCK1	*RBCK1*	RANBP2-type and C3HC4-type zinc finger containing 1	Autoinflammatory	20	NM_031229
TNFAIP3	*TNFAIP3*	TNF alpha induced protein 3	Autoinflammatory	6	NM_001270507
TNFRSF1A	*TNFRSF1A*	TNF receptor superfamily member 1A	Autoinflammatory	12	NM_001065
TRAP1	*TRAP2*	TNF receptor associated protein 1	Autoinflammatory	16	NM_016292
TRIM28	*TRIM28*	tripartite motif containing 28	Autoinflammatory	19	NM_005762
TRNT1	*TRNT1*	tRNA nucleotidyl transferase 1	Autoinflammatory	3	NM_001302946
WDR1	*WDR1*	WD repeat domain 1	Autoinflammatory	4	NM_005112
C1QA	*C1QA*	Complement component 1, q subcomponent, A chain	Complement disorders	1	NM_015991
C1QB	*C1QB*	Complement component 1, q subcomponent, B chain	Complement disorders	1	NM_000491
C1QC	*C1QC*	Complement component 1, q subcomponent, C chain	Complement disorders	1	NM_172369
C1R	*C1R*	Complement component 1r	Complement disorders	12	NM_001733
C2	*C2*	Complement component 2	Complement disorders	6	NM_000063
C3	*C3*	Complement component 3	Complement disorders	19	NM_000064
C5	*C5*	Complement component 5	Complement disorders	9	NM_001735
C6	*C6*	Complement component 6	Complement disorders	5	NM_000065
C7	*C7*	Complement component 7	Complement disorders	5	NM_000587
C8A	*C8A*	Complement component 8 alpha	Complement disorders	1	NM_000562
C8B	*C8B*	Complement component 8 beta	Complement disorders	1	NM_000066
CFH	*CFH*	Complement factor H	Complement disorders	1	NM_000186

© 2019 McCreary D et al. *JAMA Network Open.*

CFHR5	*CFHR5*	Complement factor H-related protein 5	Complement disorders	1	NM_030787
CFI	*CFI*	Complement factor 1	Complement disorders	4	NM_000204
CTPS1	*CTPS1*	CTP synthase 1	Complement disorders	1	NM_001905
MASP2	*MASP2*	Mannan binding lectin serine peptidase 2	Complement disorders	1	NM_006610
RANBP2	*RANBP2*	RAN binding protein 2	Encephalomyelitis	2	NM_006267
NLRC4	*NLRC4*	NLR family CARD domain containing 4	Haemophagocytic lymphostiocytosis	2	NM_021209
PRF1	*PRF1*	Perforin 1	Haemophagocytic lymphostiocytosis	10	NM_005041
SH2D1A	*SH2D1A*	SH2 domain containing 1A	Haemophagocytic lymphostiocytosis	X	NM_002351
SLC29A3	*SLC29A3*	Solute carrier family 29 member 3	Haemophagocytic lymphostiocytosis	10	NM_018344
STX11	*STX11*	Syntaxin 11	Haemophagocytic lymphostiocytosis	6	NM_003764
STXBP2	*STXBP2*	Syntaxin binding protein 2	Haemophagocytic lymphostiocytosis	19	NM_006949
UNC13D	*UNC13D*	Unc-13 homolog D	Haemophagocytic lymphostiocytosis	17	NM_199242
XIAP	*XIAP*	X-linked inhibitor of apoptosis	Haemophagocytic lymphostiocytosis	x	NM_001167
CLEC16A	*CLEC16A*	C-type lectin domain containing 16A	Immunodeficiency	16	NM_015226
FAS	*FAS*	Fas cell surface death receptor	Immunodeficiency	10	NM_000043
GATA2	*GATA2*	GATA binding protein 2	Immunodeficiency	3	NM_032638
IRF5	*IRF5*	Interferon regulatory factor 5	Immunodeficiency	7	NM_001098627
IRF8	*IRF8*	Interferon regulatory factor 8	Immunodeficiency	16	NM_002163
ITPR3	*ITPR3*	Inositol 1,4,5-trisphosphate receptor type 3	Immunodeficiency	6	NM_002224
MYO5A	*MYO5A*	Myosin VA	Immunodeficiency	15	NM_000259
PTPN22	*PTPN22*	Protein tyrosine phosphatase, non-receptor type 22	Immunodeficiency	1	NM_015967
RHOD	*RHOD*	Ras homolog family member D	Immunodeficiency	11	NM_014578
STAT3	*STAT3*	Signal transducer and activator of transcription 3	Immunodeficiency	17	NM_139276
STAT4	*STAT4*	Signal transducer and activator of transcription 4	Immunodeficiency	2	NM_003151

© 2019 McCreary D et al. *JAMA Network Open.*

TBK1	*TBK1*	TANK binding kinase 1	Immunodeficiency	12	NM_013254
ACP5	*ACP5*	Acid phosphatase-5/tartrate-resistant phosphatase	Interferonopathies	19	NM_001111034
ADAR	*ADAR1*	Adenosine deaminase, RNA specific	Interferonopathies	1	NM_001111
DDX58	*DDX58*	DExD/H-box helicase 58	Interferonopathies	9	NM_014314
DNASE1	*DNASE1*	Deoxyribonuclease 1	Interferonopathies	16	NM_001351825
DNASE1L3	*DNASE1L3*	Deoxyribonuclease I-like 3	Interferonopathies	3	NM_004944
DNASE2	*DNAseII*	Deoxyribonuclease 2, lysosomal	Interferonopathies	19	NM_001375
IFIH1	*IFIH1*	Interferon induced with helicase C domain 1	Interferonopathies	2	NM_022168
ISG15	*ISG15*	ISG15 ubiquitin-like modifier	Interferonopathies	1	NM_005101
NDUFS1	*NDUFS1*	NADH:ubiquinone oxidoreductase core subunit S1	White matter metabolic disease	2	NM_005006
NDUFS2	*NDUFS2*	NADH:ubiquinone oxidoreductase core subunit S2	White matter metabolic disease	1	NM_004550
NDUFS3	*NDUFS3*	NADH:ubiquinone oxidoreductase core subunit S3	White matter metabolic disease	11	NM_004551
NDUFS4	*NDUFS4*	NADH:ubiquinone oxidoreductase subunit S4	White matter metabolic disease	5	NM_002495
NDUFS5	*NDUFS5*	NADH:ubiquinone oxidoreductase subunit S5	White matter metabolic disease	1	NM_004552
NDUFS6	*NDUFS6*	NADH:ubiquinone oxidoreductase subunit S6	White matter metabolic disease	5	NM_004553
NDUFS7	*NDUFS7*	NADH:ubiquinone oxidoreductase subunit S6	White matter metabolic disease	5	NM_004553
NDUFS8	*NDUFS8*	NADH:ubiquinone oxidoreductase core subunit S8	White matter metabolic disease	11	NM_002496
POMP	*POMP*	Proteasome maturation protein	Interferonopathies	13	NM_015932
PRKCD	*PRKCD*	Protein kinase C delta	Interferonopathies	3	NM_001316327
PSMB3	*PSMB3*	Proteasome subunit beta 3	Interferonopathies	17	NM_002795
PSMB4	*PSMB4*	Proteasome subunit beta 4	Interferonopathies	1	NM_002796
PSMB8	*PSMB8*	Proteasome subunit beta 8	Interferonopathies	6	NM_148919

PSMB9	*PSMB9*	Proteasome subunit beta 9	Interferonopathies	6	NM_002800
RAB27A	*RAB27A*	RAB27A, member RAS oncogene family	Haemophagocytic lymphohistiocytosis	15	NM_004580
RNASEH2 A	*RNASEH2 A*	Ribonuclease H2 subunit A	Interferonopathies	19	NM_006397
RNASEH2 B	*RNASEH2 B*	Ribonuclease H2 subunit B	Interferonopathies	13	NM_024570
RNASEH2 C	*RNASEH2 C*	Ribonuclease H2 subunit C	Interferonopathies	11	NM_032193
SAMHD1	*SAMHD1*	SAM and HD domain containing deoxynucleoside triphosphate triphosphohydrolase 1	Interferonopathies	20	NM_015474
SKIV2L	*SKIV2L*	Ski2 like RNA helicase	Immunodeficiency	6	NM_006929
STAT2	*STAT2*	Signal transducer and activator of transcription 2	Immunodeficiency	12	NM_005419
TMEM173	*TMEM173*	Transmembrane protein 173	Interferonopathies	5	NM_198282
TREX1	*TREX1*	three prime repair exonuclease 1	Interferonopathies	3	NM_016381
USP18	*USP18*	Ubiquitin specific peptidase 18	Interferonopathies	22	NM_017414
EGR2	*EGR2*	Early growth response 2	Neuropathies	10	NM_000399
LMNA	*LMNA*	Lamin A/C	Neuropathies	1	NM_170707
LMNB1	*LMNB1*	Lamin B1	Neuropathies	5	NM_005573
GJB1	*GJB1*	Gap junction protein beta 1	Neuropathies	X	NM_000166
MFN2	*MFN2*	Mitofusin 2	Neuropathies	1	NM_014874
MPZ	*MPZ*	Myelin protein zero	Neuropathies	1	NM_000530
NEFL	*NEFL*	Neurofilament light	Neuropathies	8	NM_006158
NF1	*NF1*	Neurofibromin 1	Neuropathies	17	NM_000267
PMP22	*PMP22*	Peripheral myelin protein 22	Neuropathies	17	NM_000304
PRX	*PRX*	Periaxin	Neuropathies	19	NM_020956
TSC1	*TSC1*	TSC complex subunit 1	Neuropathies	9	NM_000368
TSC2	*TSC2*	TSC complex subunit 2	Neuropathies	16	NM_000548
TTC37	*TTC37*	Tetratricopeptide repeat domain 37	Neuropathies	5	NM_014639
LYST	*LSYT*	Lysosomal trafficking regulator	White matter and metabolic diseases	1	NM_000081
ATP7B	*ATP7B*	ATPase copper transporting beta	White matter and metabolic diseases	13	NM_000053

© 2019 McCreary D et al. *JAMA Network Open.*

CORO1A	*CORO1A*	Coronin 1A	White matter and metabolic diseases	16	NM_007074
CPT2	*CPT2*	Carnitine palmitoyltransferase 2	White matter and metabolic diseases	1	NM_000098
FOLR1	*FOLR1*	Folate receptor 1	White matter and metabolic diseases	11	NM_016725
HMBS	*HMBS*	Hydroxymethylbilane synthase	White matter and metabolic diseases	11	NM_000190
PHYH	*PHYH*	Phytanoyl-CoA 2-hydroxylase	White matter and metabolic diseases	10	NM_001037537
ARX	*ARX*	Aristaless related homeobox	White matter and metabolic diseases	X	NM_139058
ISCA2	*ISCA2*	Iron-sulfur cluster assembly 2	White matter and metabolic diseases	14	NM_194279
AARS2	*AARS2*	Alanyl-tRNA synthetase 2, mitochondrial	White matter and metabolic diseases	6	NM_020745
ABCD1	*ABCD1*	ATP binding cassette subfamily D member 1	White matter and metabolic diseases	X	NM_000033
ACOX1	*ACOX1*	Acyl-CoA oxidase 1	White matter and metabolic diseases	17	NM_001185039
ADCK3	*ADCK3*	Coenzyme Q8A	White matter and metabolic diseases	1	NM_020247
AIFM1	*AIFM1*	Apoptosis inducing factor mitochondria associated 1	White matter and metabolic diseases	X	NM_001130846
AIMP1	*AIMP1*	Aminoacyl tRNA synthetase complex interacting multifunctional protein 1	White matter and metabolic diseases	4	NM_004757
ALDH3A2	*ALDH3A2*	Aldehyde dehydrogenase 3 family member A2	White matter and metabolic diseases	17	NM_000382
ARSA	*ARSA*	Arylsulfatase A	White matter and metabolic diseases	22	NM_000487
ASPA	*ASPA*	Aspartoacylase	White matter and metabolic diseases	17	NM_000049
ATPAF2	*ATPAF2*	ATP synthase mitochondrial F1 complex assembly factor 2	White matter and metabolic diseases	17	NM_145691
BCS1L	*BCS1L*	BCS1 homolog, ubiquinol-cytochrome c reductase complex chaperone	White matter and metabolic diseases	2	NM_004328
BOLA3	*BOLA3*	BolA family member 3	White matter and metabolic diseases	2	NM_212552
BTD	*BTD*	Biotinidase	White matter and metabolic diseases	3	NM_000060

© 2019 McCreary D et al. *JAMA Network Open.*

CLCN2	*CLCN2*	Chloride voltage-gated channel 2	White matter and metabolic diseases	3	NM_001171087
COQ2	*COQ2*	Coenzyme Q2, polyprenyltransferase	White matter and metabolic diseases	4	NM_015697
COQ9	*COQ9*	Coenzyme Q9	White matter and metabolic diseases	16	NM_020312
COX10	*COX10*	Cytochrome c oxidase assembly factor heme A:farnesyltransferase	White matter and metabolic diseases	17	NM_001303
COX15	*COX 15*	Cytochrome c oxidase assembly homolog COX15	White matter and metabolic diseases	10	NM_078470
CSF1R	*CSF1R*	Colony stimulating factor 1 receptor	White matter and metabolic diseases	5	NM_005211
CST3	*CST3*	Cystatin C	White matter and metabolic diseases	20	NM_000099
CTC1	*CTC1*	CST telomere replication complex component 1	White matter and metabolic diseases	17	NM_025099
CYP27A1	*CYP27A1*	Cytochrome P450 family 27 subfamily A member 1	White matter and metabolic diseases	2	NM_000784
DARS	*DARS*	Aspartyl-tRNA synthetase	White matter and metabolic diseases	2	NM_001349
DARS2	*DARS2*	Aspartyl-tRNA synthetase 2, mitochondrial	White matter and metabolic diseases	1	NM_018122
DGUOK	*DGUOK*	Deoxyguanosine kinase	White matter and metabolic diseases	2	NM_001318859
EARS2	*EARS2*	Glutamyl-tRNA synthetase 2, mitochondrial	White matter and metabolic diseases	16	NM_133451
EIF2B1	*EIF2B1*	Eukaryotic translation initiation factor 2B subunit alpha	White matter and metabolic diseases	12	NM_001414
EIF2B2	*EIF2B2*	Eukaryotic translation initiation factor 2B subunit beta	White matter and metabolic diseases	14	NM_014239
EIF2B3	*EIF2B3*	Eukaryotic translation initiation factor 2B subunit gamma	White matter and metabolic diseases	1	NM_020365
EIF2B4	*EIF2B4*	Eukaryotic translation initiation factor 2B subunit delta	White matter and metabolic diseases	2	NM_001034116
EIF2B5	*EIF2B5*	Eukaryotic translation initiation factor 2B subunit epsilon	White matter and metabolic diseases	3	NM_003907

© 2019 McCreary D et al. *JAMA Network Open.*

ERCC2	*ERCC2*	ERCC excision repair 2, TFIIH core complex helicase subunit	White matter and metabolic diseases	19	NM_000400
ERCC3	*ERCC3*	ERCC excision repair 3, TFIIH core complex helicase subunit	White matter and metabolic diseases	2	NM_000122
ERCC6	*ERCC6*	ERCC excision repair 6, chromatin remodeling factor	White matter and metabolic diseases	10	NM_000124
ERCC8	*ERCC8*	ERCC excision repair 8, CSA ubiquitin ligase complex subunit	White matter and metabolic diseases	5	NM_000082
ETFDH	*ETFDH*	Electron transfer flavoprotein dehydrogenase	White matter and metabolic diseases	4	NM_001281737
EXOSC8	*EXOSC8*	Exosome component 8	White matter and metabolic diseases	13	NM_181503
FAM126A	*FAM126A*	Family with sequence similarity 126 member A	White matter and metabolic diseases	7	NM_032581
FUCA1	*FUCA1*	Alpha-L-fucosidase 1	White matter and metabolic diseases	1	NM_000147
FXN	*FXN*	Frataxin	White matter and metabolic diseases	9	NM_000144
GALC	*GALC*	Galactosylceramidase	White matter and metabolic diseases	14	NM_000153
GBE1	*GBE1*	1,4-alpha-glucan branching enzyme 1	White matter and metabolic diseases	3	NM_000158
GFAP	*GFAP*	Glial fibrillary acidic protein	White matter and metabolic diseases	17	NM_002055
GFM1	*GFM1*	Elongation factor mitochondrial G1	White matter and metabolic diseases	3	NM_024996
GJC2	*GJC2*	Gap junction protein gamma 2	White matter and metabolic diseases	1	NM_020435
GLA	*GLA*	Galactosidase alpha	White matter and metabolic diseases	X	NM_000169
GTF2H5	*GTF2H5*	General transcription factor IIH subunit 5	White matter and metabolic diseases	6	NM_207118
HEPACAM	*HEPACAM*	Hepatic and glial cell adhesion molecule	White matter and metabolic diseases	11	NM_152722
HFE	*HFE*	Homeostatic iron regulator	White matter and metabolic diseases	6	NM_000410
HSD17B4	*HSD17B4*	Hydroxysteroid 17-beta dehydrogenase 4	White matter and metabolic diseases	5	NM_000414
IBA57	*IBA57*	IBA57, iron-sulfur cluster assembly	White matter and metabolic diseases	1	NM_001010867
LONP1	*LONP1*	Lon peptidase 1, mitochondrial	White matter and metabolic diseases	16	NM_004793
MLC1	*MLC1*	Megalencephalic leukoencephalopathy	White matter and metabolic diseases	22	NM_015166

© 2019 McCreary D et al. *JAMA Network Open.*

		with subcortical cysts 1			
MPLKIP	*MPLKIP*	M-phase specific PLK1 interacting protein	White matter and metabolic diseases	7	NM_138701
MRPS16	*MRPS16*	Mitochondrial ribosomal protein S16	White matter and metabolic diseases	10	NM_016065
NDUFAF1	*NDUFAF1*	NADH:ubiquinone oxidoreductase complex assembly factor 1	White matter and metabolic diseases	15	NM_016013
NDUFV1	*NDUFV1*	NADH:ubiquinone oxidoreductase core subunit V1	White matter and metabolic diseases	11	NM_007103
NUBPL	*NUBPL*	Nucleotide binding protein like	White matter and metabolic diseases	14	NM_025152
PARN	*PARN*	Poly(A)-specific ribonuclease	White matter and metabolic diseases	16	NM_002582
PDHA1	*PDHA1*	Pyruvate dehydrogenase E1 alpha 1 subunit	White matter and metabolic diseases	x	NM_000284
PEX1	*PEX1*	Peroxisomal biogenesis factor 1	White matter and metabolic diseases	7	NM_000466
PEX10	*PEX10*	Peroxisomal biogenesis factor 10	White matter and metabolic diseases	1	NM_153818
PEX11A	*PEX11A*	Peroxisomal biogenesis factor 11 alpha	White matter and metabolic diseases	15	NM_003847
PEX11B	*PEX11B*	Peroxisomal biogenesis factor 11 beta	White matter and metabolic diseases	1	NM_003846
PEX12	*PEX12*	Peroxisomal biogenesis factor 12	White matter and metabolic diseases	17	NM_000286
PEX13	*PEX13*	Peroxisomal biogenesis factor 13	White matter and metabolic diseases	2	NM_002618
PEX14	*PEX14*	Peroxisomal biogenesis factor 14	White matter and metabolic diseases	1	NM_004565
PEX16	*PEX16*	Peroxisomal biogenesis factor 16	White matter and metabolic diseases	11	NM_057174
PEX2	*PEX2*	Peroxisomal biogenesis factor 2	White matter and metabolic diseases	8	NM_000318
PEX3	*PEX3*	Peroxisomal biogenesis factor 3	White matter and metabolic diseases	6	NM_003630
PEX26	*PEX26*	Peroxisomal biogenesis factor 26	White matter and metabolic diseases	22	NM_017929
PEX5	*PEX5*	Peroxisomal biogenesis factor 5	White matter and metabolic diseases	12	NM_000319
PEX6	*PEX6*	Peroxisomal biogenesis factor 6	White matter and metabolic diseases	6	NM_000287
PEX7	*PEX7*	Peroxisomal biogenesis factor 7	White matter and metabolic diseases	6	NM_000288
PGM3	*PGM3*	Phosphoglucomutase 3	White matter and metabolic diseases	6	NM_015599

© 2019 McCreary D et al. *JAMA Network Open.*

PLP1	*PLP1*	Proteolipid protein 1	White matter and metabolic diseases	x	NM_000533
POLG	*POLG*	DNA polymerase gamma, catalytic subunit	White matter and metabolic diseases	15	NM_002693
POLG2	*POLG2*	DNA polymerase gamma 2, accessory subunit	White matter and metabolic diseases	17	NM_007215
POLR1C	*POLR1C*	POLR1C	White matter and metabolic diseases	6	NM_004875
POLR3A	*POLR3A*	RNA polymerase III subunit A	White matter and metabolic diseases	10	NM_007055
POLR3B	*POLR3B*	RNA polymerase III subunit B	White matter and metabolic diseases	12	NM_018082
PSAP	*PSAP*	Prosaposin	White matter and metabolic diseases	10	NM_002778
PYCR2	*PYCR2*	Pyrroline-5-carboxylate reductase 2	White matter and metabolic diseases	1	NM_013328
RARS	*RARS*	Arginyl-tRNA synthetase	White matter and metabolic diseases	5	NM_002887
RASGRP1	*RASGRP1*	RAS guanyl releasing protein 1	White matter and metabolic diseases	15	NM_005739
RNASET2	*RNASET2*	Ribonuclease T2	White matter and metabolic diseases	6	NM_003730
RRM2B	*RRM2B*	Ribonucleotide reductase regulatory TP53 inducible subunit M2B	White matter and metabolic diseases	8	NM_001172477
SCN9A	*SCN9A*	Sodium voltage-gated channel alpha subunit 9	White matter and metabolic diseases	2	NM_002977
SCO1	*SCO1*	SCO1, cytochrome c oxidase assembly protein	White matter and metabolic diseases	17	NM_004589
SCO2	*SCO2*	SCO2, cytochrome c oxidase assembly protein	White matter and metabolic diseases	22	NM_005138
SCP2	*SCP2*	Sterol carrier protein 2	White matter and metabolic diseases	1	NM_002979
SDHAF1	*SDHAF1*	succinate dehydrogenase complex assembly factor 1	White matter and metabolic diseases	19	NM_001042631
SDHB	*SDHB*	succinate dehydrogenase complex iron sulfur subunit B	White matter and metabolic diseases	1	NM_003000
SLC16A2	*SLC16A2*	Solute carrier family 16 member 2	White matter and metabolic diseases	x	NM_006517
SLC17A5	*SLC17A5*	Solute carrier family 17 member 5	White matter and metabolic diseases	6	NM_012434
SLC1A4	*SLC1A4*	Solute carrier family 1 member 4	White matter and metabolic diseases	2	NM_003038

© 2019 McCreary D et al. *JAMA Network Open.*

SLC25A12	*SLC25A12*	Solute carrier family 25 member 12	White matter and metabolic diseases	2	NM_003705
SOX10	*SOX10*	SRY-box 10	White matter and metabolic diseases	22	NM_006941
SPTAN1	*SPTAN1*	Spectrin alpha, non-erythrocytic 1	White matter and metabolic diseases	9	NM_003127
SUCLA2	*SUCLA2*	Succinate-CoA ligase ADP-forming beta subunit	White matter and metabolic diseases	13	NM_003850
SUMF1	*SUMF1*	Sulfatase modifying factor 1	White matter and metabolic diseases	3	NM_182760
SURF1	*SURF1*	SURF1, cytochrome c oxidase assembly factor	White matter and metabolic diseases	9	NM_003172
TACO1	*TACO1*	Translational activator of cytochrome c oxidase I	White matter and metabolic diseases	17	NM_016360
TUBB4A	*TUBB4A*	Tubulin beta 4A class IVa	White matter and metabolic diseases	19	NM_006087
TUFM	*TUFM*	Tu translation elongation factor, mitochondrial	White matter and metabolic diseases	16	NM_003321
TYMP	*TYMP*	Thymidine phosphorylase	White matter and metabolic diseases	22	NM_001953
NFU1	*NFU1*	NFU1 iron-sulfur cluster scaffold	White matter and metabolic diseases	2	NM_015700
SNORD118	*SNORD118*	SMALL NUCLEOLAR RNA, C/D BOX, 118	White matter and metabolic diseases	17	NR_033294

© 2019 McCreary D et al. *JAMA Network Open.*

eTable 3. SureDesign description of probes for NIP.

MiSeq sequencing runs	NIP Design ID	NIP gene list version	Description	Number of genes	Number of regions	Number of probes	Probe size (Kbp)
Run 1	3065351	NIP 1	NIP1 genes	257	3448	23940	877.433
Run 2	3102591	NIP 1.1	NIP1 modified with more baits added to 54 regions in 28 genes and 1 region added	257	3450	24547	895.455
Run 3		NIP 1.1					
Run 4		NIP 1.1					

eTable 4. Regions below the depth of coverage threshold of 30X.

C5= complement component 5; *CFH*= complement factor H; *CFI*= complement factor 1; *CORO1A*= coronin 1A; *NF1*= neurofibromin 1; *POMP*= proteasome maturation protein; *PTPN22*= protein tyrosine phosphatase, non-receptor type 22; *RANBP2*= RANBP2-type and C3HC4-type zinc finger containing 1; *SMAD4*= SMAD family member 4; *STAT4*= signal transducer and activator of transcription 4; *TBK1*= TANK binding kinase 1; *USP18*= ubiquitin specific peptidase 18.

Gene	Chromosome	Start	Stop	Mean reads
		Coordinates	Coordinates	
C5	chr9	1.24E+08	1.24E+08	20.2813
CFH	chr1	1.97E+08	1.97E+08	28.1719
CFI	chr4	1.11E+08	1.11E+08	20.9531
CORO1A	chr16	30200112	30200352	2.53125
NF1	chr17	29548982	29549162	15.7031
NF1	chr17	29548796	29548976	26.3906
POMP	chr13	29238585	29238765	8.17188
PTPN22	chr1	1.14E+08	1.14E+08	1.03125
RANBP2	chr2	1.09E+08	1.09E+08	13.7188
RANBP2	chr2	1.09E+08	1.09E+08	14.5625
RANBP2	chr2	1.09E+08	1.09E+08	20.7031
RANBP2	chr2	1.09E+08	1.09E+08	21.9063
RANBP2	chr2	1.09E+08	1.09E+08	23.6719

© 2019 McCreary D et al. *JAMA Network Open.*

RANBP2	chr2	1.09E+08	1.09E+08	26.9375
RANBP2	chr2	1.09E+08	1.09E+08	27.8906
RANBP2	chr2	1.09E+08	1.09E+08	28.6094
SMAD4	chr18	48578898	48579018	0.73438
STAT4	chr2	1.92E+08	1.92E+08	25.8281
TBK1	chr12	64890076	64890256	3.3125
USP18	chr22	18649941	18650181	6.0625

eTable 5. List of regions with more baits added to improve coverage.

Gene	Chromosome	Start Coordinates	Stop Coordinates
C5	chr9	123744892	123745132
C5	chr9	123800134	123800314
CECR1	chr22	17700000	17700800
CECR1	chr22	17702200	17702900
CFH	chr1	196716221	196716461
CFI	chr4	110678838	110679193
CORO1A	chr16	30200112	30200352
DARS	chr2	136670005	136670185
DARS2	chr1	173814307	173814487
ERCC8	chr5	60200569	60200749
FAS	chr10	90771600	90772000
FBN2	chr5	127642770	127642950
GUCY1A3	chr4	156625024	156625204
HSD17B4	chr5	118807272	118807452
LYST	chr1	235875330	235875570
LYST	chr1	235963562	235963742
MY05A	chr15	52640928	52641108
MY05A	chr15	52688463	52688643
NF1	chr17	29541415	29541655
NF1	chr17	29548796	29548976
NF1	chr17	29548982	29549162
NF1	chr17	29579896	29580076
PARN	chr16	14678536	14678716
PEX1	chr7	92116722	92116902
PEX1	chr7	92140189	92140429

© 2019 McCreary D et al. *JAMA Network Open.*

Pomp	chr13	29238585	29238765
RANBP2	chr2	109345531	109345711
RANBP2	chr2	109347165	109347405
RANBP2	chr2	109347733	109347973
RANBP2	chr2	109363120	109363300
RANBP2	chr2	109365360	109365600
RANBP2	chr2	109367690	109367930
RANBP2	chr2	109368268	109368508
RANBP2	chr2	109369827	109370067
RANBP2	chr2	109370233	109370473
RANBP2	chr2	109371583	109371763
RANBP2	chr2	109378513	109378693
SH2D1A	chrX	123505130	123505310
SLC17A5	chr6	74345043	74345283
SMAD4	chr18	48578898	48579018
STAT4	chr2	191923375	191923555
TBK1	chr12	64853918	64854158
TBK1	chr12	64883769	64883949
TBK1	chr12	64890076	64890256
TRNT1	chr3	3186210	3186450
TTC37	chr5	94842586	94842766
USP18	chr22	18649971	18650151
USP18	chr22	18650609	18650849
USP18	chr22	18652568	18652748
USP18	chr22	18653483	18653723
USP18	chr22	18659471	18659651
XIAP	chrX	123025014	123025194

© 2019 McCreary D et al. *JAMA Network Open.*

eTable 6. Clinical features and class 4 and 5 genetic variants identified on Neuroinflammation gene panel (NIP) in patients with suspected genetic neuroinflammatory disorders and unknown molecular diagnoses. MRI=magnetic resonance imaging; GAD=glutamic acid decarboxylase; OGB=oligoclonal bands; JAK=janus kinase; HSCT=haematopoietic stem cell transplantation; HLH=haemophagocytic lymphostiocytosis; ANA=antinuclear; IVIG=intravenous immunoglobulin; CT=computed tomography; CSF=cerebrospinal fluid; AD=autosomal dominant; AR=autosomal recessive. T=tolerated; B=benign; D=damaging; N=polymorphism; Het= heterozygous; Hom= Homozygous; Hemi= hemizygosity; XLR= X-linked recessive; XL= X-linked.

Patient number	Sex	Age (Years)	Gene	Nucleotide Change	Amino Acid Change	Predicted pathogenicity	Mode of inheritance/ Zygosity	Variant Classification	Clinical Features, laboratory investigations and treatment	Clinical impact of NIP and suspected diagnosis after genetic testing
17	F	18	*NLRP3*	c.2557A>G	p.Thr853Ala	T/B/D	AD/Het	3	**Clinical features:** Recurrent episodes of encephalopathy, seizures, psychiatric symptoms, left homonymous quantranopia, diabetes, autoimmune thyroiditis. No other systemic symptoms or other organ involvement. Relapsing disease course. **Investigations:** MRI brain showed abnormal signal and atrophic changes in right occipital lobe with extension in right thalamus and mesiotemporal lobe. Atrophic changes subsequently extended to right cerebellar hemisphere and right parietal lobe. Positive GAD antibodies, negative other autoantibodies. No acute phase response. **Treatment:** IVIG	Unclassified neuroinflammatory disease
			NLRC4	c.2785G>T	p.Ala929Ser	T/B/.	AD/Het	3		
			RANBP2	c.7172C>G	p.Thr2391Ser	T/B/N	AD/Het	3		
			FBN2	c.7181T>C	p.Ile2394Thr	T/B/N	AD/Het	3		
			STX11	c.9C>A	p.Asp3Glu	D/D/D	AR/Het	3		
			TSC1	c.2194C>T	p.His732Tyr	D/P/A	AD/Het	4		
			NOTCH1	c.4028C>T	p.Ala1343Val	T/P/D	AD/Het	3		
			NOTCH1	c.1981G>A	p.Gly661Ser	T/P/D	AD/Het	3		
			POLG	c.158_159insGCA	p.Gln55_Pro56insGln	./././.	AD;AR/Het	3		
			MEFV	c.442G>C	p.Glu148Gln	D/D/P	AD;AR/Het	3		
			RNF213	c.4156G>A	p.Asp1386Asn	T/B/N	-/Het	3		
			DNASE2	c.134C>G	p.Ala45Gly	T/P/N	-/Het	3		

© 2019 McCreary D et al. *JAMA Network Open.*

18	M	15	***ADAR***	c.577C>G	p.Pro193Ala	D/D/D	AR;AD/Het	5	**Clinical features:** Encephalitis triggered by influenza A, cutaneous leucocytoclastic vasculitis, peripheral lymphoedema. **Investigations:** MRI brain showed bilateral haemorhhagic foci and brain oedema. Negative autoantibodies, normal complement studies, modestly elevated ESR (20 mm/h; RR< 10 mm/h). **Treatment:** Corticosteroids and azathioprine.	Likely monogenic interferonopathy.
			CFHR5	c.832G>A	p.Gly278Ser	D/D/N	AD/Het	3		
			TGFBR2	c.374del	p.K128Sfs*34	./././	AD/Het	3		
			PEX1	c.3250A>G	p.Met1084Val	T/P/D	AR/Het	3		
			SPTAN1	c.1303T>G	p.Ser435Ala	T/B/D	AD/Het	3		
			POLG	c.158_159insGCA	p.Gln55_Pro56insGln	./././	AR;AD/Het	3		
			RNF213	c.5162C>T	p.Pro1721Leu	T/./N	-/Het	3		
			LPIN2	c.1801G>A	p.Glu601Lys	T/B/N	-/Het	3		
19	M	14	*FUCA1*	c.106C>T	p.Arg36Cys	D/P/D	AR/Het	3	**Clinical features:** Recurrent acute transverse myelitis, optic neuritis, hearing loss, choreoathetosis, development delay. No other systemic symptoms or other organ involvement. Chronic relapsing disease course. **Investigations:** MRI brain showed bilateral and symmetrical T1 hyperintense signal in the globi pallidi, bilateral mild engorgement of the deep medullary veins (more on the left) with	Unclassified neuroinflammatory disease
			BTD	c.133G>A	p.Gly45Arg	T/B/N	AR/Het	4		
			PEX1	c.1579A>G	p.Thr527Ala	T/B/D	AR/Het	3		
			CLEC16A	c.2945G>A	p.Ser982Asn	D/D/D	-/Het	3		
			POLG	c.158_159insGCAGCA	p.Gln55_Pro56insGlnGln	./././	AD;AR/Hom	3		
			LONP1	c.10A>G	p.Ser4Gly	T/B/D	AR/Het	3		
			MEFV	c.442G>C	p.Glu148Gln	D/D/P	AD;AR/Het	3		
			HSD17B4	c.272G>A	p.Gly91Glu	D/D/D	AR/Het	3		

© 2019 McCreary D et al. *JAMA Network Open.*

									some associated haemorrhagic foci, generalised brain parenchymal swelling. Negative autoantibodies and CSF OGB negative. No acute phase response. **Treatment:** IVIG, corticosteroids, rituximab	
20	F	3	***DARS***	c.1391C>T	p.Pro464Leu	T/D/D	AR/Het	4	**Clinical features:** Subcutaneous nodules, progressive ataxia, muscle weakness, lipodystrophy. **Investigations:** MRI brain revealed widespread leucoencephalopathy. Negative autoantibody screen and no acute phase response. Significant upregulation of interferon induced gene expression. **Treatment:** corticosteroids, oral JAK1/JAK2 inhibitor, mycophenolate mofetil..	Likely monogenic interferonopathy
			PRX	c.1574T>C	p.Val525Ala	T/B/N	AD;AR/Het	3		
			PRX	c.133C>G	p.Arg45Gly	D/P/D	AD;AR/Het	3		
			CBS	c.833T>C	p.Ile278Thr	D/D/A	AR/Het	3		
			FBN2	c.6598A>G	p.Met2200Val	T/B/D	AD/Het	3		
			MYH11	c.3949C>A	p.Leu1317Ile	T/P/D	AD/Het	3		
			NDUFS3	c.475G>C	p.Val159Leu	T/B/D	AR/Het	3		
			PLCG2	c.2542C>T	p.Leu848Phe	T/D/D	AD/Het	3		
			PRKG1	c.1421A>T	p.Tyr474Phe	D/B/D	AD/Het	3		
			RNF213	c.5114C>A	p.Thr1705Lys	D/./D	-/Het	3		
			TNFAIP3	c.1105G>A	p.Ala369Thr	T/B/N	AD/Het	3		
21	M	8	*IL1RN*	c.379G>A	p.Ala127Thr	T/B/N	AD;AR/Het	3	**Clinical features:** Febrile episode followed by refractory seizures, progressive four limb motor	Autoimmune lymphoproliferative syndrome.
			LONP1	c.70C>A	p.Leu24Met	D/D/N	AR/Het	3		

© 2019 McCreary D et al. *JAMA Network Open.*

			MFN2	c.58C>T	p.His20Tyr	T/B/D	AD;AR/Het	3	dystonic disorder following infectious gastroenteritis. History of frequent chest infections.<br>**Investigations:** MRI brain showed marked atrophy and signal changes followed by cystic degeneration on subsequent scans in basal ganglia, internal capsule and midbrain. Negative autoantibody screen and no acute phase response.<br>**Treatment:** Corticosteroids	Referred to Immunology for consideration of HSCT.
			PEX13	c.661C>G	p.Leu221Val	T/B/D	AR/Het	3		
			POLR3A	c.3499G>C	p.Val1167Leu	T/P/D	AR/Het	3		
			TGFBI	c.C829C>G	p.Leu277Val	T/B/D	AD/Het	3		
			USP18	c. 974A>G	p.Asn325Ser	T/B/N	AR/Het	3		
			COL3A1	c.1804C>A	p.Pro602Thr	T/P/D	AD/Het	3		
			TMEM173	c.937G>A	p.Ala313Thr	T/B/N	AD/Het	3		
			C5	c.3051G>C	p.Glu1017Asp	D/D/D	AD/Het	3		
			FAS	c.517G>A	p.Glu173Lys	T/B/N	AD/Het	4		
22	M	18	*ERCC3*	c.2080G>A	p.Ala694Thr	T/B/D	AR/Het	3	**Clinical features:** Recurrent seizures, progressive ataxia, dysarthria, loss of mobility, learning difficulties, necrotising pneumonia.<br>**Investigations:** MRI brain changes suggestive of chronic lymphocytic inflammation with pontine perivascular enhancement responsive to steroids (CLIPPERS) syndrome. ANA/dsDNA and complement studies normal. Blood film no	Primary HLH diagnosed posthumously
			BTD	c.1171C>T	p.Pro391Ser	T/B/N	AR/Het	3		
			ERCC8	c.170G>A	p.Arg57Lys	T/B/D	AR/Het	3		
			FBN2	c.976C>T	p.Pro326Ser	T/B/D	AD/Hom	3		
			RRM2B	c.44C>T	p.Pro15L	./././.	AD;AR/Het	3		
			PRF1	c.1621del	p.M541Cfs*71	./././.	AR/Het	4		
			PRF1	c.82C>T	p.Arg28Cys	D/P/N	AR/Het	4		
			NUBPL	c.545T>C	p.Val182Ala	D/P/D	AR/Het	3		

© 2019 McCreary D et al. *JAMA Network Open.*

			MYO5A	c.5065G>A	p.Val1689Ile	T/B/D	AR/Het	3	lymphocyte vaculation. Muscle biopsy and other metabolic disease tests- normal. Brain biopsy chronic white matter disease with dense macrophage infiltrate. History of occasional cytopaenias. **Treatment:** IVIG, mycophenolate mofetil, azathioprine, rituximab, natalizumab.	
			POLG	c.158_159insGCA	p.Gln55_Pro56insGln	./././.	AD;AR/Het	3		
			TSC2	c.4524_4526del	p.Phe1510del	./././.	AD/Het	3		
			MYH11	c.4156A>G	p.Thr1386Ala	T/B/D	AD/Het	3		
			CTC1	c.974A>G	p.Ser83Thr	D/D/D	AR/Het	3		
23	F	12	***DGUOK***	c.462T>A	p.Asn154Lys	D/D/D	AR/Het	4	**Clinical features:** Facial lipodystrophy, recurrent seizures, progressive myoclonus, occasional fevers, no rashes or articular symptoms. **Investigations:** MRI brain no progressive white matter disease. Negative autoantibody screen. **Treatment:** rituximab, mycophenolate mofetil, IVIG and methotrexate.	Unclassified neuroinflammatory disease
			MVK	c.1129G>A	p.Val377Ile	T/B/A	AD;AR/Het	4		
			RANBP2	c.6362A>G	p.Gln2121Arg	T/D/D	AD/Het	3		
			RANBP2	c.6680A>G	p.Tyr2227Cys	D/D/D	AD/Het	3		
			TGFBR2	c.374del	p.Lys128Sfs*34	./././.	AD/Het	3		
			CFI	c.1657C>T	p.Pro553Ser	T/B/N	AD;AR/Het	3		
			PRF1	c.755A>G	p.Asn252Ser	T/B/N	AR/Het	3		
			PLCG2	c.82A>T	p.Met28Leu	T/P/D	AD/Het	3		
			IRF8	c.724T>C	p.Tyr242His	D/P/D	AD/Het	3		
			RNF213	c.1208_1210del	p.Gly405del	./././.	-/Het	3		
			USP18	c.974A>G	p.Asn325Ser	T/B/N	AR/Het	3		

© 2019 McCreary D et al. *JAMA Network Open.*

24	F	10	*YY1AP1*	c.516G>C	p.Met172Ile	T/D/D	AR/Het	3	**Clinical features:** Right sided visual loss secondary to long standing optic disc swelling and optic neuritis. No systemic symptoms or other organ involvement. **Investigations:** MRI brain scan, nonprogressive white matter disease (non-consistent with MS). Negative autoantibody profile and no acute phase response. **Treatment:** corticosteroids.	Unclassified neuroinflammatory disease
			LYST	c.1686G>C	p.Gln562His	D/P/D	AR/Het	3		
			RANBP2	c.7172C>G	p.Thr2391Ser	T/B/N	AD/Het	3		
			PEX1	c.2528G>A	p.Gly843Asp	D/D/A	AR/Het	4		
			ATPAF2	c.511G>A	p.Val171Met	T/B/D	AR/Het	3		
			GFAP	c.650G>A	p.Arg217Gln	T/B/D	AD/Het	3		
			TRIM28	c.2183CT	p.Thr728Ile	T/B/N	AD/Het	3		
			RBCK1	c.703G>A	p.Glu235Lys	T/B/D	AR/Het	3		
			USP18	c.506C>T	p.Thr169Met	T/B/N	AR/Het	3		
			ARSA	c.*96A>G	-	./././.	AR/Het	3		
			ARSA	c.1055A>G	p.Asn352Ser	T/B/P	AR/Het	3		
25	F	15	***ATP7B***	c.3207C>A	p.His1069Gln	D/D/A	AR/Het	4	**Clinical features:** Progressive polyneuropathy, glaucoma, spinal cord tethering, atrial septal defect, ventricular septal defect, proptosis, precocious telarche, keratosis pilaris. **Investigations:** MRI brain and spine showed diffuse thickening of the nerve roots with Meckel's	Unclassified neuroinflammatory disease
			MASP2	c.1243G>A	p.Asp415Asn	T/B/D	AR/Het	3		
			CPT2	c.1763C>G	p.Ser588Cys	D/D/D	AD;AR/Het	3		
			LYST	c.5518T>G	p.Ser1840Ala	T/B/D	AR/Het	3		
			DGUOK	c.4G>T	p.Ala2Ser	T/B/N	AR/Het	3		
			RANBP2	c.6362A>G	p.Gln2121Arg	T/D/D	AD/Het	3		

© 2019 McCreary D et al. *JAMA Network Open.*

			RANBP2	c.6680A>G	p.Tyr2227Cys	D/D/D	AD/Het	3	caves, foramina ovale and the jugular foramina, the cauda equina, in all spinal exit foramina, the lumbar plexus and the right sciatic nerve. Negative autoantibody profile and no acute phase response. Negative CSF OGB and normal levels of CSF neopterins. **Treatment:** corticosteroids and IVIG.	
			TGFBR2	c.374del	p.Lys128Sfs*34	./././.	AD/Het	3		
			MYLK	c.3706A>G	p.Met1236Val	T/B/N	AD/Het	3		
			GFM1	c.1775A>T	p.Xaa592Leu	././N	AR/Het	3		
			AIMP1	c.7C>T	p.Prp3Ser	D/./N	AR/Het	3		
			C6	c.2435C>T	p.Thr812Ile	D/D/D	-/Het	3		
			C6	c.1555G>A	p.Asp519Asn	D/D/D	-/Het	3		
			TGFBI	c.1954G>C	p.Glu652Gln	D/B/D	AD/Het	3		
			POLR1C	c.944C>T	p.Ala315Val	D/P/N	AR/Het	3		
			NEFL	c.1576G>A	p.Glu526Lys	./B/.	AD;AR/Het	3		
			ERCC6	c.3659C>T	p.Thr1220Ile	T/B/N	AD;AR/Het	3		
			GALC	c.1403C>G	p.Thr468Ser	D/D/D	AR/Het	3		
			POLG	c.58_159insGCAGCA	p.Gln55_Pro56insGlnGln	././.	AD;AR/Het	3		
			POLG	c.125G>A	p.Arg42Gln	T/B/N	AD;AR/Het	3		
			TSC2	c.3914C>T	p.Pro1305Leu	T/B/N	AD/Het	3		
			MEFV	c.586G>T	p.Gly196Trp	D/B/N	AD;AR/Het	3		

© 2019 McCreary D et al. *JAMA Network Open.*

			NOD2	c.1622G>A	p.Arg541Gln	T/B/N	AD;Mu/Het	3		
26	M	3	***SH2D1A***	c.163C>T	p.Arg55Xaa	./A	XLR/Het	5	**Clinical features:** Seizures, acute lethargy, incoherent speech, aphasia, neutropaenia. Chronic relapsing disease course. **Investigations:** MRI brain showed widespread bilateral white matter disease progressive over time with brain volume loss. Brain biopsy showed non-specific lymphocytic infiltrate. Negative autoantibody profile and no acute phase response. Negative CSF OGB **Treatment:** IVIG, steroids, cyclophosphamide. plasma exchange.	X-linked lymphoproliferative disease. Underwent allogeneic HSCT. Sibling diagnosed with XLP-type 1 and underwent HSCT too.
			RANBP2	c.6362A>G	p.Gln2121Arg	T/D/D	AD/Het	3		
			RANBP2	c.7172C>G	p.Thr2391Ser	T/B/N	AD/Het	3		
			C6	c.542C>T	p.Thr181Ile	T/B/N	-/Het	3		
			NOTCH1	c.3664G>A	p.Val1222Met	T/B/D	AD/Het	3		
			POLR3A	c.1745G>T	p.Arg582Leu	D/B/D	AR/Het	3		
			DNASE1	c.105G>C	p.Glu35Asp	T/B/D	AD/Het	3		
			NLRP12	c.1855G>C	p.Glu619Gln	D/D/D	AD/Het	3		
			CBS;CBSL	c.1105C>T	p.Arg369Cys	D/D/D	AR/Het	3		
			USP18	c.1115G>T	p.Cys372Phe	D/B/N	AR/Het	3		
27	F	17months	*SKI*	c.1163C>T	p.Ala388Val	T/B/N	AD/Het	3	**Clinical features:** Necrotising encephalopathy with seizures leading to death preceded by chest infection. **Investigations**: MRI brain showed oedema, no focal lesions. Negative autoantibody profile, cytopaenias, no acute phase response.	Unclassified neuroinflammatory disease
			RANBP2	c.7172C>G	p.Thr2391Ser	T/B/N	AD/Het	3		
			BTD	c.1171C>T	p.Pro391Ser	T/B/N	AR/Het	3		
			TGFBR2	c.374del	p.Lys128SERfs*34	./.	AD/Het	3		
			COQ2	c.64A>T	p.Arg22Xaa	./A	AD;AR/Het	3		

© 2019 McCreary D et al. *JAMA Network Open.*

			MEFV	c.1772T>C	p.Ile591Thr	T/B/N	AD;AR/Het	3	Negative CSF OGB. **Treatment:** corticosteroids and IVIG, plasma exchange with no response.	
			COX10	c.1291C>T	p.Arg431Trp	D/P/N	AR;Mi/Het	3		
			STXBP2	c.1034C>T	p.Thr345Mey	D/D/D	-/Het	4		
			PRF1	c.272C>T	p.Ala91Val	D/D/D	AR/Het	3		
			USP18	c.1097T>C	p.Val366Ala	D/P/N	AR/Het	3		
			TRNT1	c.1057-7C>G	p.intronic	./../.	AR/Het	5		
28	M	8	*ISG15*	c.388C>T	p.Pro130Ser	T/P/N	AR/Het	3	**Clinical features:** Refractory epilepsy. Mild skin rashes occasional fevers. Chronic relapsing disease course. **Investigations:** Brain MRI showed white matter disease in the left temporal pole, both mesial frontal lobes, left insular, left peritrigonal and parietal regions with diffuse cortical atrophy particularly in the left frontal and perirolandic regions. Brain biopsy showed chronic inflammatory process affecting both white and grey matter with no fibrinoid necrosis of leukocytoclasia.	Muckle Wells syndrome (Cryopyrin associated periodic syndrome). Referred to rheumatology for consideration of starting IL-1 blockade therapy.
			PEX10	c.611G>A	p.Arg204His	T/B/N	AR/Het	3		
			C8A	c.1331G>A	p.Arg444His	D/D/D	AR/Het	3		
			NLRP3	c.214G>A	p.Val72Met	D/D/N	AD/Het	4		
			RANBP2	c.1258A>G	p.Thr420Ala	T/B/N	AD/Het	3		
			ERCC3	c.847C>T	p.Arg283Cys	D/P/D	AR/Het	3		
			CYP27A1	c.491G>C	p.Arg164Pro	D/D/D	AR/Het	3		
			GATA2	c.595G>A	p.Gly199Arg	D/D/D	AD/Het	3		
			ITPR3	c.4364G>A	p.Arg1455His	T/D/D	AR/Het	3		
			SAMD3	c.731del	p.Leu244Yfs*16	./../.	-/Het	3		
			FAM126A	c.1171G>C	p.Gly391Arg	D/B/D	AR/Het	3		

© 2019 McCreary D et al. *JAMA Network Open.*

			ERCC6	c.4315G>C	p.Ala14 39Pro	D/D/D	AD;AR/He t	3	Positive CSF OGB and elevated neopterins 211 nmol/l (RR< 65 nmol/). Negative antibodies for Myelin Oligodendrocyte Glycoprotein /Aquaporin 4/ ANA/dsDNA. **Treatment:** Corticosteroids and mycophenolate mofetil.	
			STAT2	c.2478G>T	p.Gln82 6His	D/B/N	AR/Het	3		
			PMP22	c.353C>T	p.Thr11 8Met	D/D/A	AD;AR/He t	4		
			NOTCH3	c.1490C>T	p.Ser49 7Leu	T/B/D	AD/Het	3		
			SLC2A10	c.1057C>G	p.Leu35 3Val	T/B/N	AR/Het	3		
29	M	15	*NDUFS2*	c.1004A>T	p.Glu33 5Val	D/P/P	AR/Het	3	**Clinical features:** Recurrent episodes of occipital headache, bilateral arm weakness, visual symptoms, collapse. **Investigations:** MRI brain showed patchy bilateral a nd asymmetric white matt er lesions in the cerebral hemispheres and posterior fossa with some attenuation of the posterior circulation vesse ls on MRA. Catheter angiography cerebral and visceral normal. No spinal pathology. Brain biopsy showed multiple foci of chronic inflammation comprising predominantly T-lymphocytes, present in the cortex, white matter and	Primary HLH
			RANBP2	c.5756G>A	p.Gly19 19Asp	T/B/B	AD/Het	3		
			RANBP2	c.6362A>G	p.Gln21 21Arg	T/D/D	AD/Het	3		
			RANBP2	c.6680A>G	p.Tyr22 27Cys	D/D/D	AD/Hom	3		
			RANBP2	c.7172C>G	p.Thr23 91Ser	T/B/B	AD/Het	3		
			RANBP2	c.7669A>G	p.Asn25 57Asp	T/B/B	AD/Het	3		
			TGFBR2	c.449del	p.Lys15 3Serfs* 34	./././.	AD/Hom	3		
			FBN2	c.8351C>T	p.Pro27 84Leu	T/B/B	AD/Het	3		
			CSF1R	c.2891_289 2insC	p.Gln96 5Alafs* 11	./././.	AD/Hom	3		
			PEX6	c.2219T>C	p.Leu74 0Pro	D/D/D	AD;AR/He t	3		
			PRF1	c.731T>G	p.Leu24 4Arg	D/D/D	AR/Het	4		

© 2019 McCreary D et al. *JAMA Network Open.*

			PRF1	c.694C>T	p.Arg232Cys	D/D/D	AR/Het	4	possibly on leptomeningeal surface in perivascular but no obvious evidence of vasculitis.<br>Occasional foci show features of possible early granulomas, but there is no necrosis. RNA sequencing for identification of any pathogens on brain biopsy tissue was negative. Tuberculosis PCR and extended culture negative .Abnormal perforin expression and NK cell killing activity. CSF OGB negative; raised neopterins 211 (RR < 65 nmol/L). Normal complement studies, negative autoantibodies including MOG/Aq4. Mannose binding lectin deficient.<br>**Treatment:** Corticosteroids.	
			PSTPIP1	c.79C>T	p.R27W	D/./.	AD/Het	3		
			DNASE1	c.460C>G	p.P154A	D/B/B	AD/Het	3		
			MEFV	c.442G>C	p.E148Q	D/D/P	AD;AR/Het	3		
			USP18	c.974A>G	p.N325S	T/B/B	AR/Het	3		
			DARS2	c.228-21T>C	p.intronic	./. /.	AR/Hom	4		
			DARS2	c. 228-20_-21insC	p.intronic	./. /.	AR/Het	4		
30	F	12	***CFHR5***	c.Ala480dup	p.Glu163Argfs*34	./. /.	AD/Hom	4	**Clinical features:** Short history of fever and headache followed by rapid deterioration of conscious level and evidence raised ICP. No systemic symptoms or other organ involvement.	Unclassified neuroinflammatory disease
			CFHR5	c.622T>C	p.Cys208Arg	D/D/N	AD/Het	3		
			LYST	c.8806G>A	p.Val2936Ile	T/B/D	AR/Het	3		
			RANBP2	c.7172C>G	p.Thr2391Ser	T/B/N	AD/Het	3		

© 2019 McCreary D et al. *JAMA Network Open.*

			CFI	c.1555G>A	p.Asp519Asn	D/D/D	AD;AR/Het	3	Bilateral papillitis and/or papilloedema.<br>**Investigations:** MRI brain suggested fulminant presumed ADEM.<br>Biopsy – no vasculitis, monocytic infiltrate, no marked histochemical evidence of demyelination.<br>Negative autoantibody profile and no acute phase response.<br>Negative CSF OGB.<br>**Treatment:** Required EVD and decompressive craniectomy.	
			CFI	c.1019T>C	p.Ile340Thr	D/P/N	AD;AR/Het	3		
			PEX7	c.377A>C	p.Gln126Pro	T/P/D	AR/Het	3		
			GALC	c.334A>G	p.Thr112Ala	T/D/D	AR/Het	4		
			NDUFAF1	c.958G>A	p.Glu320Lys	T/B/N	AR/Het	3		
			POLG	c.158_159insGCA	p.Gln55_Pro56insGln	./././.	AD;AR/Hom	3		
			C3	c.4855A>C	p.Ser1619Arg	T/P/N	AD;AR/Het	3		
			TRIM28	c.1180G>C	p.Asp394His	D/D/D	AD/Het	3		
			USP18	c.974A>G	p.Asn325Ser	T/B/N	AR/Het	3		
31	F	8	*PEX10*	c.435G>A	p.Met145Ile	T/B/N	AR/Het	3	**Clinical features:** Severe progressive retinal ischaemia and retinal angiopathy.<br>**Investigations**: MRI brain non-specific stable white matter disease.<br>Negative autoantibody profile. No acute phase response.<br>**Treatment:** Nil	POLG2 related metabolic disorder. Referral to metabolic team and further metabolic test investigation were organised.
			C8B	c.1505C>T	p.Ser502Phe	D/D/D	AR/Het	3		
			YY1AP1	c.1157T>C	p.Ile386Thr	D/B/B	AR/Het	3		
			HSD17B4	c.1192T>C	p.Ser398Pro	T/B/D	AR/Het	3		
			PSMB8	c.220A>T	p.Thr74Ser	D/D/D	AR/Het	3		
			SPTAN1	c.1688T>C	p.Met563Thr	T/B/D	AD/Het	3		
			SLC29A3	c.7G>A	p.Val3Ile	T/B/N	AR/Het	3		

© 2019 McCreary D et al. *JAMA Network Open.*

			PEX16	c.760G>C	p.Val254Leu	T/B/N	AR/Het	3		
			POLG2	c.1105A>G	p.Arg369Gly	D/D/D	AD/Hom	4		
			NLRP12	c.2222A>G	p.Asn741Ser	T/B/N	AD/Het	3		
			MEFV	c.442G>C	p.Glu148Gln	D/D/P	AD;AR/Het	3		
			USP18	c.974A>G	p.Asn325Ser	T/B/N	AR/Het	3		
32	F	10	*C8A*	c.1454G>A	p.Arg485His	D/B/P	AR/Het	3	**Clinical features:** Recurrent fevers with acute phase response, arthralgia, aseptic meningitis. **Investigations:** MRI brain showed non progressive white matter disease. Negative autoantibody profile and no systemic symptoms or other organ involvement. **Treatment:** corticosteroids.	Autoinflammation, antibody deficiency, and immune dysregulation syndrome (APLAID). Family screening, consideration of IL-1 blockade.
			CLCN2	c.704G>A	p.Arg235Gln	D/D/D	AD;AR/Het	3		
			PEX7	c.377A>C	p.Gln126Pro	T/P/D	AR/Het	3		
			MPLKIP	c.253G>C	p.Gln85Arg	D/B/N	AR/Het	3		
			ELN	c.1861G>A	p.Ala621Thr	D/B/N	AD/Het	3		
			TNFRSF1A	c.1343C>T	p.Prp448Leu	T/B/N	AD/Het	3		
			STAT2	c.2473G>T	p.Gly825Cys	T/B/N	AR/Het	3		
			POLG	c.158_159insGCA	p.Gln55_Pro56insGln	./././.	AD;AR/Het	3		
			POLG	c.156_158del	p.Gln55del	./././.	AD;AR/Het	3		
			POLG	c.125G>A	p.Arg42Gln	T/B/N	AD;AR/Het	3		
			PLCG2	c.1444T>C	p.Tyr482His	T/B/D	AD/Het	4		

© 2019 McCreary D et al. *JAMA Network Open.*

			PLCG2	c.1712A>G	p.Asn571Ser	D/B/D	AD/Het	4		
			CBS;CBSL	c.833T>C	p.Ile278Thr	D/D/A	AR/Het	4		
			PEX26	c.728C>T	p.Ala243Val	T/B/N	AR/Het	3		
			USP18	c.1097T>C	p.Val366Ala	D/D/N	AR/Het	3		
			USP18	c.1115G>T	p.Cys372Phe	D/B/N	AR/Het	3		
33	M	9	*C8A*	c.1724C>T	p.P575L	T/B/P	AR/Het	3	**Clinical features:** Left sided squint and vision loss. Left sided hemiplegia. No systemic symptoms or other organ involvement.<br>**Investigations:** MRI brain showed bilateral symmetrical cortical and subcortical lesions in the parieto occipital lesions affecting both grey and white matter disease with associated oedema and haemorhhagic transformation.<br>Brain biopsy suggested chronic inflammatory disorder with perivascular component.<br>Negative CSF OGB.<br>Negative autoantibody profile.<br>Primary HLH functional	Folate receptor deficiency
			NDUFS2	c.58C>A	p.P20T	D/B/P	AR/Het	3		
			LYST	c.8368A>C	p.K2790Q	D/B/P	AR/Het	4		
			RANBP2	c.8842A>C	p.S2948R	T/P/N	AD/Het	3		
			TGFBR2	c.374del	p.Lys128Serfs*34	./././.	AD/Hom	3		
			CFI	c.1217G>A	p.Arg406His	T/P/P	AD;AR/Het	3		
			OTULIN	c.439C>T	p.Pro147Ser	T/B/N	AR/Het	3		
			FBN2	c.2260G>A	p.Gly754Ser	T/P/D	AD/Het	3		
			TGFBI	c.805C>T	p.Leu269Phe	D/D/D	AD/Het	3		
			PHYH	c.85C>T	p.Pro29Ser	T/B/P	AR/Het	3		
			GALC	c.49G>A	p.Ala17Thr	T/./P	AR/Het	3		
			NDUFAF1	c.941C>G	p.Ala314Gly	D/P/P	AR/Het	3		

© 2019 McCreary D et al. *JAMA Network Open.*

			POLG	c.158_159insGCA	p.Gln55_Pro56insGln	./. /.	AD;AR/Hom	3	assays normal.<br>**Treatment:** corticosteroids and cyclophosphamide, mycophenolate mofetil.	
			TRAP1	c.2053G>A	p.Asp685Asn	T/D/D	-/Het	3		
			GFAP	c.883G>A	p.Asp295Asn	D/D/P	AD/Het	3		
			MEFV	c.442G>C	p.Glu148Gln	D/D/P	AD;AR/Het	3		
			PRX	c.944G>A	p.Arg315Gln	T/B/D	AD;AR/Het	3		
			MLC1	c.628G>A	p.Val210Ile	T/B/D	AR/Het	3		
			FOLR1	c.493+2T>C	p.intronic	./. /.	AR/Hom	4		
34	F	21	*DARS2*	c.1257G>A	p.Met419Ile	T/B/N	AR/Het	3	**Clinical features:** Chorea. No other symptoms or organ involvement.<br>**Imaging:** MRI brain showed progressive white matter disease. Autoantibody screen negative and infections as trigger excluded.<br>**Treatment:** IVIG, corticosteroids, mycophenolate mofetil.	Unclassified neuroinflammatory disease
			NOTCH1	c.3836G>A	p.Arg1279His	T/B/D	AD/Het	3		
			EXOSC8	c.815G>C	p.Ser272Thr	T/P/D	AR/Het	5		
			POLG	c.156_158del	p.Gln55del	./. /.	AD;AR/Hom	3		
			GFAP	c.1288C>T	p.Arg430Cys	D/P/D	AD/Het	3		
			POLG2	c.562G>C	p.Gly188Arg	D/D/D	AD/Het	3		
			PRX	c.1568T>C	p.Leu523Pro	T/B/N	AD;AR/Het	3		
			PRX	c.1483G>C	p.Glu495Gln	T/B/D	AD;AR/Het	3		
			USP18	c.974A>G	p.Asn325Ser	T/B/N	AR/Het	3		

© 2019 McCreary D et al. *JAMA Network Open.*

35	M	9	***STXBP2***	c.1034C>T	p.Thr345Met	D/D/D	AD;AR/het	4	**Clinical features:** Enterocolitis, headache, cognitive decline, HLH. **Investigations:** MRI brain showed white matter disease non progressive. Negative autoantibody screen and no acute phase response. **Treatment:** Corticosteroids, IVIG, ciclosporin	Unclassified neuroinflammatory disease with HLH
			MFN2	c.58C>T	p.His20Tyr	T/B/D	AR/Het	3		
			YY1AP1	c.169C>T	p.Leu57Phe	T/D/D	AD/Hom	3		
			RANBP2	c.7499C>T	p.Thr2500Ile	D/B/N	AR/Het	3		
			SKIV2L	c.3279G>C	p.Glu1093Asp	D/D/D	AR/Het	3		
			NOTCH1	c.4129C>T	p.Pro1377Ser	T/B/N	AD/Het	3		
			PLCG2	c.1712A>G	p.Asn571Ser	D/B/D	AD/Het	3		
			CTC1	c.2162G>C	p.Gly72Ala	T/B/N	AR/Het	3		
			CTC1	c.1241G>C	p.Gly414Ala	T/D/D	AR/Het	3		
36	M	1	***RNASEH2B***	c.179T>G	p.Leu60Arg	D/D/A	AR/Het	5	**Clinical features:** Neonatal meningoencephalitis, recurrent seizures, development delay. **Investigations:** MRI brain showed meningoencephalitis. CSF lymphocytosis and raised neopterins (105 nmol/lRR< 65 nmol/l), bilateral widespread white matter disease, normal XIAP expression, negative screening for congenital infection. Negative autoantibody profile.	Aicardi Goutières syndrome (AGS) type 2. Family screening, consideration of JAK inhibition treatment
			RNASEH2B	c.529G>A	p.Ala177Ter	T/D/A	AR/Het	5		
			SDHB	c.487T>C	p.Ser163Pro	T/B/A	AD/Het	3		
			C8B	c.1625C>T	p.Thr542Ile	T/B/N	AR/Het	3		
			NLRC4	c.1163G>A	p.Arg388Gln	T/B/.	AD/Het	3		
			RANBP2	c.5941T>A	p.Ser1981Thr	T/B/N	AD/Het	3		
			CFI	c.1322A>G	p.Lys441Arg	T/B/N	AD;AR/Het	3		
			ITPR3	c.626A>G	p.Glu209Gly	D/D/D	AR/Het	3		

© 2019 McCreary D et al. *JAMA Network Open.*

			PEX16	c.760G>C	p.Val254Leu	T/B/N	AR/Het	3	Abnormal liver function tests.<br>**Treatment:** Corticosteroids	
			POLG	c.158_159insGCAGCA	p.Gln55_Pro56insGlnGln	./. /.	AD;AR/Hom	3		
			POLG	c.125G>A	p.Arg42Gln	T/B/N	AD;AR/Het	3		
			DNASE1	c.503C>T	p.Ala168Val	D/P/D	AD/Het	3		
			PRX	c.4077_4079del	p.Glu1361del	./. /.	AD;AR/Het	3		
			XIAP	c.1429G>A	p.Ala477Thr	D/D/D	XLR/Hemi	3		
37	F	10	***SCN9A***	c.2215A>G	p.Ile739Val	D/P/D	AD;AR/Het	4	**Clinical features:** Opsoclonus myoclonus and neuroblastoma in infancy, lupus like disease with recurrent arthritis, cutaneous lesions, malar rash.<br>**Investigations:** MRI brain widespread white matter disease. Positive ANA and dsDNA, Aq4 antibodies.<br>**Treatment:** corticosteroids and mycophenolate mofetil.	Unclassified neuroinflammatory disease
			CFHR5	c.136C>T	p.Pro46Ser	T/B/N	AD/Het	3		
			SCN9A	c.3734A>G	p.Asn1245Ser	D/D/D	AD;AR/Het	3		
			DDX58	c.212G>A	p.Arg71His	D/D/D	AD/Het	3		
			STAT2	c.685G>A	p.Glu229Lys	T/D/D	AR/Het	3		
			ATP7B	c.751C>T	p.His251Tyr	T/B/N	AR/Het	3		
			TRAP1	c.1073A>G	p.Glu358Gly	T/B/N	-/Het	3		
			GFAP	c.1288C>T	p.Arg430Cys	D/P/D	AD/Het	3		
			RNF213	c.6551A>G	p.Gln2184Arg	T/B/N	-/Het	3		
			LPIN2	c.1801G>A	p.Glu601Lys	T/B/D	-/Het	3		

© 2019 McCreary D et al. *JAMA Network Open.*

			PRX	c.716C>T	p.Pro239Leu	T/P/D	AD;AR/Het	3		
			NEFL	c.1414-1C>T	p.intronic	./././.	-/Hom	3		
38	M	17	*PLOD1*	c.1217C>T	p.Pro406Leu	D/D/D	AR/Het	3	**Clinical features:** Lupus like disease with skin rashes and arthralgia. **Investigations:** MRI brain showed progressive white matter disease, intracerebral calcification. Positive dsDNA antibodies. **Treatment:** corticosteroids.	Unclassified neuroinflammatory disease
			RANBP2	c.5941T>A	p.Ser1981Thr	T/B/N	AD/Het	3		
			ERCC3	c.144G>C	p.Glu48Asp	T/B/D	AR/Het	3		
			COQ2	c.64A>T	p.Arg22Xaa	././A	AD;AR/Het	3		
			CSF1R	c.1237G>A	p.Gly413Ser	T/P/N	AD/Het	3		
			HFE	c.193A>T	p.Ser65Cys	D/D/A	AR/Het	4		
			SLC17A5	c.1375G>A	p.Val459Met	D/D/D	AR/Het	3		
			PEX1	c.2953C>T	c.Gky1220Thr	D/D/D	AR/Het	3		
			SUCLA2	c.1220G>T	p.Arg407Leu	D/D/D	AR/Het	3		
			POLG	c.2492A>G	p.Tyr831Cys	T/D/A	AD;AR/Het	3		
			POLG	c.158_166del	p.Gln53_Gln55del	././.	AD;AR/Het	3		
			NOD2	c.2722G>C	p.Gly908Arg	D/D/D	AR;Mu/Het	3		
			POLG2	c.1247G>C	p.Gly416Ala	T/D/D	AD/Het	3		
			RNF213	c.11465G>T	p.Arg3822Ile	D/D/D	-/Het	3		

© 2019 McCreary D et al. *JAMA Network Open.*

			RNASEH2A	c.615T>A	p.Asp205Glu	T/B/D	AR/Het	3		
39	F	8	***TRNT1***	c.667A>G	p.Ile223Val	T/P/D	AR/Het	5	**Clinical features:** Right sided hemiplegia, blurred vision, pyrexia, cytopaenias, seizures. No systemic symptoms or other organ involvement. Chronic progressive disease course. **Investigations:** CT diffuse cerebral oedema and swelling, MRI brain white matter disease. Negative autoantibody profile. Negative CSF OGB. **Treatment:** corticosteroids.	Unclassified neuroinflammatory disease
			RANBP2	c.2351G>A	p.Arg784Lys	T/B/N	AD/Het	3		
			NDUFS1	c.29T>C	p.Leu10Pro	D/B/N	AR/Het	3		
			CYP27A1	c.673C>T	p.Arg225Cys	D/D/D	AR/Het	3		
			MYLK	c.2101G>A	p.Ala701Thr	T/B/N	AD/Het	3		
			MYLK	c.1684G>A	p.Glu562Lys	D/P/D	AD/Het	3		
			TTC37	c.860A>G	p.Lys287Arg	T/B/D	AR/Het	3		
			FAM126A	c.1555T>C	p.Ser519Pro	D/P/D	AR/Het	3		
			NOTCH1	c.4451A>G	p.Asn1484Ser	T/B/D	AD/Het	3		
			MYO5A	c.5018C>T	p.Ser1673Leu	D/P/D	AR/Het	3		
			PSTPIP1	c.80G>A	p.Arg27Gln	D/./N	AD/Het	3		
			POLG	c.803G>C	p.Gly268Ala	D/D/D	AD;AR/Het	3		
			POLG	c.153_158del	p.Gln54_Gln55del	./././.	AD;AR/Het	3		
			PARN	c.1102A>T	p.Ser368Cys	D/P/D	AD;AR/Het	3		
			MYH11	c.5819C>A	p.Pro1940Gln	D/B/D	AD/Het	3		

© 2019 McCreary D et al. *JAMA Network Open.*

			NOD2	c.781G>T	p.Ala261Ser	T/B/N	AD;Mu/Het	3		
			RNF213	c.103G>A	p.Glu35Lys	T/B/N	-/Het	3		
			RNF213	c.7417G>A	p.Ala2473Thr	T/B/N	-/Het	3		
			DNASE2	c.611A>G	p.His204Arg	T/B/N	-/Het	3		
			CST3	c.88G>A	p.Gly30Ser	T/B/N	AD/Het	3		
			PEX26	c.130C>T	p.Leu44Phe	T/B/N	AR/Het	3		
			USP18	c.389G>A	p.Cys130Tyr	T/B/N	AR/Het	3		
			NEFL	4345+2C>T	p.Intronic	./././.	AD;AR/Hom	3		
40	M	6	***STXBP2***	c.1621G>A	p.Gly541Ser	D/D/D	AR/Het	5	**Clinical features:** Progressive ataxia, headaches, encephalopathy. No systemic symptoms or other organ involvement. **Investigations:** MRI brain showed progressive cerebellar white matter disease and dentate changes associated with cerebellar swelling and crowding of posterior fossa. Elevated CSF neopterins 95 (RR< 65), negative CSF OGB, normal ESR and CRP. Negative autoantibody profile. Abnormal T-cell	Primary HLH. Referred to immunology and undergoing work up for HSCT
			STXBP2	c.1247-1G>C	p.Intronic	././D	AR/Het	5		
			PLOD1	c.555G>T	p.Lys185Asn	T/B/D	AR/Het	3		
			PEX11B	c.13G>A	p.Val5Ile	./././.	AR/Het	3		
			COQ8A	c.11T>A	p.Ile4Lys	T/D/D	AR/Het	3		
			LYST	c.3499G>A	p.Val1167Ile	T/B/N	AR/Het	3		
			RANBP2	c.5941T>A	p.Ser1981Thr	T/B/N	AD/Het	3		
			IFIH1	c.2863C>G	p.Gln955Glu	T/B/N	AD/Het	3		
			CSF1R	c.533G>C	p.Ser178Thr	T/B/N	AD/Het	3		

© 2019 McCreary D et al. *JAMA Network Open.*

			COL4A1	c.C161C>T	p.Pro54Leu	D/D/D	AD/Het	3	and NK cell degranulation assay.<br>**Treatment:** mycophenolate mofetil, corticosteroids	
			SAMHD1	c.G1583G>A	p.Arg528Lys	T/B/N	AD;AR/Het	3		
41	F	7	***IFIH1***	c.2336G>A	p.Arg779His	T/D/D	AD/Het	5	**Clinical features:** Severe developmental delay, past medical history of opsoclonus myoclonus and neuroblastoma, lupus like disease characterised by cutaneous vasculitis and a malar rash.<br>**Investigations:** MRI brain showed wide spread white matter disease.<br>Raised inflammatory markers and positive autoantibodies (ANA, anti-dsDNA).<br>**Treatment:** mycophenolate mofetil, corticosteroids	AGS type 7.<br>Started treatment with JAK inhibitor.<br>Family counselling and genetic screening.
			C2	c.1109C>T	p.Ala370Val	T/B/N	AR/Het	3		
			TSC1	c.2285A>G	p.Asn762Ser	T/B/D	AD/Het	3		
			POLG	c.158_159insGCA	p.Gln55_Pro56insGln	./././.	AD;AR/Het	3		
			CLEC16A	c.2287G>A	p.Val763Met	T/D/D	-/Het	3		
			ACOX1	c.628C>T	p.Arg210Cys	D/D/D	AR/Het	3		
			NEFL	4345+2C>T	p.Intronic	./././.	AD;AR/Hom	3		
42	F	13	***RAB27A***	c.550C>T	p.Arg184Xaa	././D	AR/Het	5	**Clinical features**: Strabismus, ataxia. Mild erythematous skin rashes, no fevers or other systemic symptoms.<br>**Investigations:** MRI brain widespread progressive white matter disease in pons medulla and cerebellum.<br>Negative autoantibody profile. No cytopaenias.	Griscelli syndrome.<br>Referred to immunology and undergoing work up for HSCT
			RAB27A	c.259G>C	p.Ala87Pro	D/D/A	AR/Het	5		
			PEX10	c.880A>G	p.Thr294Ala	T/D/D	AR/Het	3		
			DGUOK	c.125C>T	p.Ser42Phe	D/D/D	AR/Het	3		
			DGUOK	c.218A>G	p.Gln73Arg	D/D/D	AR/Het	3		
			ITPR3	c.626A>G	p.Glu209Gly	D/D/D	AR/Het	3		

© 2019 McCreary D et al. *JAMA Network Open.*

			SAMD3	c.149T>C	p.Ile50Thr	D/D/D	-/Het	3	Negative CSF OGB. Abnormal T-cell and NK cell degranulation assay. **Treatment:** mycophenolate mofetil, corticosteroids	
			UNC13D	c.2243C>T	p.Ala748Val	T/P/D	-/Het	3		
			TRIM28	c.1073A>C	p.N358T	D/B/D	AD/Het	3		

© 2019 McCreary D et al. *JAMA Network Open.*

eTable 7. Clinical features and class 3 genetic variants identified on Neuroinflammation gene panel (NIP) in 34 patients with suspected genetic neuroinflammatory disorders and unknown molecular diagnoses. MRI=magnetic resonance imaging; GAD=glutamic acid decarboxylase; OGB=oligoclonal bands; JAK=janus kinase; HSCT=haematopoietic stem cell transplantation; HLH=haemophagocytic lymphohistiocytosis; ANA=antinuclear; IVIG=intravenous immunoglobulin; CT=computed tomography; CSF=cerebrospinal fluid; AD=autosomal dominant; AR=autosomal recessive; Mu= mutation; SMu= somatic mutation; XL= X-linked; XLR= X-linked recessive; T=tolerated; B=benign; D=damaging; N=polymorphism; Het= heterozygous; Hom= Homozygous; Hemi= hemizygosity; ESR=erythrocyte sedimentation rate; CRP=C-reactive protein.

Patient number		Sex	Age (Years)	Gene	Nucleotide Change	Amino Acid Change	Predicted pathogenicity	Mode of inheritance/ Zygosity	Variant Classification	Clinical Features, investigations and treatment	Clinical impact of NIP and suspected diagnosis after genetic testing
43		F	19	*C7*	c.289A>G	p.Ile97Val	T/D/D	-/Het	3	**Clinical features**: Chromosome 6p24-26 deletion syndrome, progressive motor difficulties, colobomas, retinal changes, and scoliosis. **Investigations:** Symmetrical white matter disease, ventriculomegaly. Negative autoantibody screen and no systemic symptoms or other organ involvement. No acute phase response. Negative CSG OGB and normal levels of CSF neopterins. **Treatment:** corticosteroids and	Unclassified neuroinflammatory disease
				FBN2	C.362A>G	p.Asp121Gly	D/P/D	AD/Het	3		
				NOTCH1	c.4823G>A	p.Arg1608His	T/D/D	AD/Het	3		
				HFE	c.283G>C	p. Asp 95His	D/D/D	AD/AR/Het	3		
				NOD2	c.859C>T	p.His287Tyr	T/P/N	AD/Het	3		
				NOD2	c.2046G>T	p. Leu 682Phe	D/D/D	AD/ Het	3		
				RANBP2	c.6362A>G	p. Gln2121Arg	T/D/D	AD/Het	3		
				RANBP2	c.7172C>G	p.Thr2391Ser	T/B/N	AD/Het	3		
				RARS	c.982G>A	p. Val 328 Ile	T/B/N	AR/Het	3		
				USP18	c.974A>G	p.Asn325Ser	T/B/N	AR/Het	3		

© 2019 McCreary D et al. *JAMA Network Open.*

				USP18	c.1115G>T	p.Cys372Phe	D/B/N	AR/Het	3	mycophenolate mofetil. Required VP shunt for ventriculomegaly.	
44		F	15	*MASP2*	c.881C>T	p.Thr294Met	D/B/N	AR/Het	3	**Clinical features**: Ophthalmoplegia, proptosis, retinal artery occlusion. No systemic symptoms or other organ involvement. **Investigations:** MRI brain showed widespread leucoencephalopathy. Mitochondrial DNA mutation testing negative. Negative autoantibody screen and no acute phase response. **Treatment:** Nil	Unclassified neuroinflammatory disease
				PLOD1	c.1534C>T	p.Arg512Cys	D/D/D	AR/Het	3		
				COPA	c.766G>C	p.Val256Leu	T/B/D	AD/Het	3		
				PRF1	c.632C>T	p.Ala211Val	T/B/N	AR/Het	3		
				PLCG2	c.2011A>G	p.Ile671Val	T/B/D	AD/Het	3		
				PRX	c.4376C>T	p.Ala1459Val	D/D/D	AD;AR/Het	3		
				RANBP2	c.3253A>C	p.Lys1085Gln	T/D/D	AD/Het	3		
				SLC2A10	c.674G>A	p.Arg225His	D/D/N	AR/Het	3		
				USP18	c.974A>G	p.Asn325Ser	T/B/N	AR/Het	3		
				C2	c.1305T>A	p.His435Gln	D/D/D	AR/Het	3		
				MEFV	c.442G>C	p.Glu148Gln	D/D/P	AD;AR/Het	3		
				ELN	c.1492G>A	p.Ala498Thr	D/D/D	AD/Het	3		
				SPTAN1	c.4525G>A	p.Asp1509Asn	T/B/D	AD/Het	3		
45		F	9	*PEX14*	c.26A>T	p.Gln9Leu	D/B/D	AR/Het	3	**Clinical features:** HLH, febrile seizures, serositis, enteropathy, hypothyroidism	Likely primary HLH
				C1QC	c.559G>A	p.Val187Ile	T/B/N	AR/Het	3		

				ERCC3	c.1411G>A	p.Val471Ile	D/D/D	AR/Het	3	**Investigations:** bilateral symmetrical white matter lesions, extensive infection screening negative, primary HLH functional assays negative. **Treatment:** corticosteroids, anakinra, methotrexate, baricitinib, plasma exchange, IVIG.	
				MYLK	c.1182_1184del	p.Val395del	./././.	AD/Hom	3		
				CLCN2	c.1859A>T	p.Glu620Val	D/B/D	AD;AR/Het	3		
				C6	c.2800G>A	p.Ala934Thr	T/B/N	-/Het	3		
				C6	c.1848G>A	p.Met616Ile	D/B/N	-/Het	3		
				C6	c.1701G>C	p.Gln567His	T/B/N	-/Het	3		
				NDUFS4	c.373G>T	p.Val125Phe	T/P/N	AR;Mi;XLD/Het	3		
				CSF1R	c.733G>A	p.Ala245Thr	T/B/N	AD/Het	3		
				PARN	c.1741G>A	p.Gly581Arg	T/B/N	AD;AR/Het	3		
				NOD2	c.1411C>T	p.ARG471Cys	T/B/N	AD;Mi/Het	3		
				CTC1	c.370T>G	p.Leu124Val	T/B/N	AR/Het	3		
				STXBP2	c.953C>T	p.Thr318Met	T/D/D	-/Het	3		
				STXBP2	c.1375C>T	p.Arg459Tyr	D/D/N	-/Het	3		
				NOTCH3	c.5764G>T	p.Val1922Leu	T/D/D	AD/Het	3		
				PRX	c.2101G>A	p.Val701M	T/P/N	AD;AR/Het	3		
				CBS;CBSL	c.588G>C	p.Arg196Ser	T/B/D	AR/Het	3		
				USP18	c.974A>G	p.Asn325Ser	T/B/N	AR/Het	3		

© 2019 McCreary D et al. *JAMA Network Open.*

46		F	8months	*PSMB9*	c.517C>T	p.Arg173Cys	D/D/D	AR/Het	3	**Clinical features**: Seizures, developmental delay, Right sided hemiplegia and lethargy, livedo reticularis. No other systemic symptoms or other organ involvement.<br>**Investigations:** MRI brain showed left sided MCA infarct and widespread white matter disease.<br>Skin biopsy suggested occlusive endotheliopathy.<br>Negative autoantibody screen and no acute phase response.<br>**Treatment:** corticosteroids and aspirin, mycophenolate mofetil.	Unclassified neuroinflammatory disease
				SKIV2L	c.1705G>A	p.Val569Met	T/D/D	AR/Het	3		
				RANBP2	c.1258A>G	p.Thr420Ala	T/B/N	AD/Het	3		
				PEX6	c.235G>C	p.Ala79Pro	T/B/N	AR/Het	3		
				ISG15	c.142C>T	p.His48Tyr	T/B/N	AR/Het	3		
				RNF213	c.12463A>C	p.Thr4155Pro	T/P/N	AR/Het	3		
				PRF1	c.272C>T	p.Ala91Val	D/D/D	AR/Het	3		
				USP18	c.974A>G	p.Asn325Ser	T/B/N	AR/Het	3		
47		F	15	*C8A*	c.1558C>T	p.Arg520Cys	D/D/N	AR/Het	3	**Clinical features**: Progressive neurological disorder with no systemic symptoms or other organ involvement.<br>**Investigations:** MRI brain showed cystsic encephalomalacia affecting left frontal lobe, right occipital lobe and right temporal lobe. Meningeal biopsy showed	Unclassified neuroinflammatory disease
				ELN	c.1363G>A	p.Ala455Thr	././N	AD/Het	3		
				GATA2	c.481C>G	p.Pro161Ala	T/D/D	AD/Het	3		
				NOD2	c.1292C>T	p.Ser431Leu	D/P/N	AD/Het	3		
				NOD2	c.1390G>A	p. Gly 464Arg	T/P/N	AD/Het	3		
				NOD2	c.2377G>A	p.Val793Met	D/P/D	AD/Het	3		

© 2019 McCreary D et al. *JAMA Network Open.*

				PEX26	c.911G>A	p.Arg304 His	T/B/N	AR/Het	3	lymphocytic infiltrate and reactive gliosis. Autoantibodies negative. Negative CSF OGB. **Treatment:** corticosteroids, mycophenolate mofetil.	
				PHYH	c.238C>T	p.Arg80C ys	T/B/D	AR/Het	3		
				RNASE T2	c.643G>A	p.Glu215 Lys	T/D/N	AR/Het	3		
				TSC2	c.3140T>C	p. Val 1047Ala	T/B/D	AD/Het	3		
				TUFM	c.760G>A	p. Ala 254Thr	T/B/N	AR/Het	3		
				USP18	c.974A>G	p.Asn325 Ser	T/B/N	AR/Het	3		
				WDR1	c.512C>T	p.Ala171 Val	T/B/D	-/Het	3		
48		F	14	*ATP7B*	c.442C>T	p.Arg148 Trp	D/D/D	AR/Het	3	**Clinical features**: Spasticity, dystonia, sensory neuropathy, severe myopia and astigmatism, episode of right sided hemiparesis and collapse. No systemic symptoms or other organ involvement. **Investigations:** MRI/MRA brain showed subacute right intraventricular haemorrhage and a left MCA infarct with involvement of the basal ganglia associated with cerebral oedema. MRA showed irregular narrowing of the left ICA and distal basilar	Unclassified neuroinflammatory disease
				C7	c.1606G>A	p.Val536I le	T/B/N	-/Het	3		
				COL4A 1	c.1673C>T	p.Ala558 Val	T/B/D	AD/Het	3		
				FBN1	c.5865G>T	p.Gln195 5His	T/B/D	AD/Het	3		
				HEPAC AM	c.139G>A	p.Val47M et	T/D/D	AR/Het	3		
				LPIN2	c.1876C>T	p.Pro626 Ser	T/B/N	-/Het	3		
				NDUFS 2	c.1234G>C	p.Val412 Leu	D/B/D	AR/Het	3		
				USP18	c.974A>G	p.Asn325 Ser	T/B/N	AR/Het	3		

© 2019 McCreary D et al. *JAMA Network Open.*

										artery.<br>Negative CSF OGB and normal CSF neopterins. Negative autoantibody screen and no acute phase response.<br>**Treatment:** corticosteroids	
49		M	8	*ATP7B*	c.4175T>C	p.Met1392Thr	T/B/D	AR/Het	3	**Clinical features**: Severe bilateral optic atrophy and visual impairment, bulbar palsy, hearing loss. No systemic symptoms or other organ involvement.<br>**Investigations:** MRI brain showed extensive dural thickening most prominently along the right frontal convexity with cavernous sinus involvement and narrowing of the left ICA, signal abnormality associated with extensive dural enhancement along the left cerebellar hemisphere and obliteration of the left sigmoid sinus where it drains into the internal jugular vein on the left, consistent with jugular vein thrombosis.	Likely STA-4 deficiency, novel disease on-going functional experiments
				C5	c.4133A>T	p.Tyr1378Phe	T/B/N	AD/Het	3		
				C6	c.11G>T	p.Arg4Leu	T/B/N	-/Het	3		
				CBL	c.105_106insCAC	p.His42_L43insHis	./././.	AD;Smu/Hom	3		
				CBS	c.1273G>A	p.Val425Met	D/D/D	AR/Het	3		
				CFHR5	c.427A>C	p.Thr143Pro	T/P/N	AD/Het	3		
				COL3A1	c.130G>A	p. Val44Ile	T/D/D	AD/Het	3		
				COQ8A	c.1876C>T	p.Arg626Cys	T/B/N	AR/Het	3		
				COX10	c.773T>A	p. Leu258His	D/D/D	AR; MI/Het	3		
				CSF1R	c.721A>G	p.N241Asp	T/B/N	AD/Het	3		
				CYP27A1	c.506C>T	p. Ala169Val	T/P/N	AR/Het	3		
				ELN	c.931G>A	p.Ala311Thr	D/D/D	AD/Het	3		
				FBN2	c.4784C>T	p.Thr1595 Ile	T/B/N	AD/Het	3		

© 2019 McCreary D et al. *JAMA Network Open.*

				FOXP3	c.171_173del	p.Ser60del	./././	AR;XLR/Hom	3	Necrotising granulomatous inflammation on brain biopsy. Negative autoantibodies. normal ACE, no infection identified (including TB and atypical TB). **Treatment:** anti TB medication, corticosteroids and azathioprine.	
				ITPR3	c.1588G>C	p.Gly530Arg	T/D/D	AR/Het	3		
				NOTCH1	c.6454G>C	p.Gly2152Arg	T/B/	AD/Het	3		
				PEX1	c.1142C>A	p.Ala381Asp	D/B/N	AR/Het	3		
				PHYH	c.568G>A	p. Val190Ile	T/D/D	AR/Het	3		
				POLG	c.158_159insGCA	p.Gln55_P56insGln	./././	AD;AR/Het	3		
				RNF213	c.103G>A	p.Glu35Lys	T/B/N	-/Het	3		
				SLC29A3	c.74G>A	p.Arg25Gln	T/B/N	AR/Het	3		
				STAT4	Exon 4 Deletion	-	-	-/Hom	-		
				TMEM173	c.659G>A	p.Arg220His	T/B/N	AD/Het	3		
				TSC2	c.4316G>A	p.Gly1439Asp	T/P/N	AD/Het	3		
				TTC37	c.4414A>G	p.Thr1472Ala	T/B/N	AR/Het	3		
				UNC13D	c.740T>G	p.Val247Gly	D/D/D	-/Het	3		
				USP18	c.974A>G	p.Asn325Ser	T/B/N	AR/Het	3		
				USP18	c.1089T>A	p.Tyr363Xaa	././D	AR/Het	3		
50		M	14	*ADAR*	c.1577A>G	p.Gln526Arg	D/D/D	AD;AR/Het	3	**Clinical features**: Systemic lupus	

© 2019 McCreary D et al. *JAMA Network Open.*

				CTC1	c.1795C>T	p.Pro599 Ser	T/B/N	AR/Het	3	erythematosus disease with skin rashes, arthralgia. **Investigations:** positive ANA and dsDNA antibodies, with worsening leucoencephalopathy on MRI brain imaging, but no intracerebral calcification. **Treatment:** corticosteroids, cyclophosphamide and rituximab.	Likely systemic lupus erythematosus
				MASP2	c.1351G>A	p.Ala451 Thr	T/B/P	AR/Het	3		
				MFN2	c.439C>A	p.Leu147I le	T/B/D	AD;AR/Het	3		
				MYLK	c.1007C>T	p.Pro336 Leu	D/P/N	AD/Het	3		
				PSTPIP 1	c.203C>T	p.Thr68M et	T/P/D	AD/Het	3		
				RANBP 2	c.2339C>T	p.Pro780 Leu	D/B/D	AD/Het	3		
				RANBP 2	c.6362A>G	p. Gln2121 Arg	T/D/D	AD/Het	3		
				RNF213	c.15017C>T	p.Ser5006 Leu	T/B/N	-/Het	3		
				TGFBR 2	c.374delA	p.Lys128 Serfs*34	./././.	AD/Hom	3		
				UNC13 D	c.2782C>T	p.Arg928 Cys	T/B/N	-/Het	3		
51		F	10	*C5*	c.512T>C	p.Val171 Ala	D/D/D	AD/Het	3	**Clinical features**: Acute onset hemiplegia and encephalopalthy. No systemic symptoms or other organ involvement. **Investigations:** MRI/MRA showed bilateral multifocal vasculopathy. Negative autoantibody screen and no acute phase	Unclassified neuroinflamma tory disease
				C7	c.1258A>C	p.Lys420 Gln	D/D/D	-/Het	3		
				C8B	c.1625C>T	p.Thr542I le	T/B/N	AR/Het	3		
				CBS	c.518T>C	p.Ile173T hr	D/D/A	AR/Het	4		
				CLEC16 A	c.2728G>A	p.Gly910 Ser	T/P/D	-/Het	3		
				EIF2B3	c.407A>G	p.Gln136 Arg	T/B/N	AR/Het	3		

© 2019 McCreary D et al. *JAMA Network Open.*

				ELN	c.328A>g	p.Ala110 Thr	D/D/D	AD/Het	3	response. Negative CSF OGB.<br>**Treatment:** Corticosteroids, IVIG	
				GJC2	c.1067A>G	p.Gln356 Arg	T/B/N	AD;AR/Het	3		
				RANBP 2	c.7754T>C	p.Ile2585 Thr	T/B/N	AD/Het	3		
				RNF213	c.6551A>G	p.Gln218 4Arg	T/B/N	AD/Het	3		
				TGFBR 2	c.374delA	p.Lys128 Serfs*34	./././.	AD/Hom	3		
				TSC2	c.856A>G	p.Met286 Val	T/B/N	AD/Het	3		
				USP18	c.295G>A	p.Val99Il e	T/D/N	AR/Het	3		
				USP18	c.974A>G	p.Asn325 Ser	T/B/N	AR/Het	3		
				USP18	c.1115G>T	p.Cys372 Phe	D/B/N	AR/Het	3		
				GLA	c.61C>T	p.Leu21P he	T/B/N	XL/Het	3		
52		M	3	*AARS2*	c.1661G>A	p.Arg554 His	T/B/D	AR/Het	3	**Clinical features**: Encephalopathy<br>**Investigations:** MRI brain suggestive of diffuse necrotising encephalopathy clinical presentation. No systemic symptoms or other organ involvement. Positive ANA and ACL antibodies at presentation subsequently negative when tested 12 weeks later.	Unclassified neuroinflammatory disease
				EIF2B3	c.1025C>T	p.Ser342 Leu	T/B/N	AR/Het	3		
				LPIN2	c.755C>T	p.Ala252 Val	T/B/N	-/Het	3		
				MEFV	c.442G>C	p.Glu148 Gln	D/D/P	AD;AR/Het	3		
				SLC25A 12	c.995T>G	p.Leu332 Arg	D/D/D	AR/Het	3		

										**Treatment:** corticosteroids and IVIG.	
53		F	2	*C2*	c.839_849del	p.Met280 Asnfs*3	./. /.	AR/Het	3	**Clinical features**: Fever, maculopapular rash, encephalopathy, anaemia, lymphadenopathy, hepatosplenomegaly. **Investigations:** MRI brain showed diffuse oedema. Positive CSF OGB. Negative autoantibodies and extensive infection screen negative. **Treatment:** corticosteroids, IVIG, plasma exchange.	Unclassified neuroinflamma tory disease
				C5	c.2653C>T	p.Arg885 Cys	T/P/N	AD/Het	3		
				C5	c.1060C>A	p. Leu 354Met	D/D/D	AD/Het	3		
				CYP27A 1	c.536A>G	p.Asn179 Ser	D/P/D	AR/Het	3		
				CYP27A 1	c.1151C>T	p.Pro384 Leu	D/D/D	AR/Het	3		
				NDUFA F1	c.199G>A	p.Val67Il e	D/B/N	AR/Het	3		
				PEX10	c.880A>G	p.Thr294 Ala	T/D/D	AR/Het	3		
				PEX2	c.748T>C	p.Trp250 Arg	T/D/D	AR/Het	3		
				PYCR2	c.898C>G	p.Leu300 Val	D/B/N	AR/Het	3		
				SAMD3	c.1378G>A	p.Asp460 Asn	T/D/D	-/Het	3		
				SLC1A4	c.40G>A	p.Ala14Tj r	T/B/D	AR/Het	3		
				STAT2	c.2478G>T	p.Gln826 His	D/B/N	AR/Het	3		
				TRAP1	c.1432_1433 insCCG	p.Ser477_ Gly 478insAla	./. /.	-/Het	3		
				TRAP1	c.1429T>G	p.Ser477 Ala	T/B/N	-/Het	3		
				UNC13 D	c.2782C>T	p.Arg928 Cys	T/B/N	-/Het	3		

© 2019 McCreary D et al. *JAMA Network Open.*

				USP18	c.974A>G	p.Asn325 Ser	T/B/N	AR/Het	3		
54		M	11	*C8B*	c.444C>G	p.Asp148 Glu	D/D/D	AR/Het	3	**Clinical features**: Febrile induced epilepsy refractory to treatment. **Investigations:** MRI brain showed symmetrical contrast enhancing basal ganglia and external capsule, cerebral cortex and frontal lobe lesions associated with brain atrophy. Normal spinal appearances. Extensive metabolic, infectious disease and rheumatological causes excluded. **Treatment:** Corticosteroids	Unclassified neuroinflammatory disease
				RANBP 2	c.6362A>G	p.Gln212 1Arg	T/D/D	AD/Het	3		
				C6	c.1355G>A	p. Gly 452Glu	D/B/N	-/Het	3		
				C6	c.10C>T	p.Arg4Cy s	T/B/N	-/Het	3		
				PEX1	c.1579A>G	p.Thr527 Ala	T/B/D	AR/Het	3		
				PEX2	c.748T>C	p.Trp250 Arg	T/D/D	AR/Het	3		
				TRAP1	c.598A>G / c.757A>G	p.Ile200V al / Ile253Val	T/B/D	-/Het	3		
				PEX26	c.457C>G	p.Leu153 Val	T/B/N	AR/Het	3		
55		F	7	*EIF2B4*	c.913C>G	p.Arg305 Gly	T/B/N	AR/Het	3	**Clinical features**: Spasticity, dystonia, bulbar symptoms with dysphagia, speech regression, microcephaly. No systemic symptoms or other organ involvement. **Investigations:** MRI brain showed lack of myelin maturation in frontal lobes, no leukodystrophy. Negative autoantibody screen and no systemic	Unclassified neuroinflammatory disease
				NLRC4	c.2785G>T	p.Ala929 Ser	T/B/.	AD/Het	3		
				COL3A 1	c.505C>T	p.Leu169 Phe	T/B/N	AD/Het	3		
				CFI	c.1322A>G	p.Lys441 Arg	T/B/N	AD;AR/Het	3		
				ETFDH	c.572G>A	p.Gly191 Asp	D/P/D	AR/Het	3		
				OTULI N	c.345G>T	p.Gln115 His	T/B/D	AR/Het	3		
				C2	c.1922T>C	p.Val641 Ala	T/B/N	AR/Het	3		

© 2019 McCreary D et al. *JAMA Network Open.*

				SPTAN1	c.3803T>C	p.Val1268Ala	D/D/D	AR/Het	3	symptoms or other organ involvement. **Treatment**: IVIG.	
				MEFV	c.1222C>T	p.Arg408Trp	T/B/N	AD;AR/Het	3		
				ALDH3A2	c.1270C>T	p.Pro424Ser	T/B/D	AR/Het	3		
				GFAP	c.1288C>T	p.Arg430Cys	D/B/D	AD/Het	3		
				USP18	c.1115G>T	p.Cys372Phe	D/B/N	AR/Het	3		
56		M	11	*EIF2B5*	c.1202G>A	p.Arg401Gln	T/B/N	AR/Het	3	**Clinical features**: Encephalopathy, seizures, cognitive dysfunction. No systemic symptoms or other organ involvement. **Investigations:** MRI brain showed diffuse cortical blurring with symmetrical signal abnormality in the claustra adjacent to the external capsules and the juxtacortical white matter of the right temporal lobe. No spinal pathology. Negative autoantibody profile and no acute phase response. **Treatment:** corticosteroids and IVIG.	Unclassified neuroinflammatory disease
				CLCN2	c.218G>A	p.Arg73His	T/B/D	AD;AR/Het	3		
				TTC37	c.3808C>G	p.Pro1270Ala	T/B/D	AR/Het	3		
				RARS	c.982G>A	p.Val328Ile	T/B/N	AR/Het	3		
				GALC	c.17A>G	p.His6Arg	D/B/N	AR/Het	3		
				NDUFAF1	c.215C>G	p.Thr72Ser	T/B/N	AR/Het	3		
				RNF213	c.14999A>G	p.Gln5000Arg	D/D/D	-/Het	3		
				USP18	c.295G>A	p.Val99Ile	T/P/N	AR/Het	3		
				MEFV	c.442G>C	p.Glu148Gln	D/D/P	AD;AR/Het	3		
				ARSA	c.*96A>G	-	./././.	AR/Het	3		
				ARSA	c.1055A>G	p.Asn352Ser	T/B/P	AR/Het	3		
57		F	13	*LYST*	c.8913T>G	p.Asn2971Lys	T/P/D	AR/Het	3	**Clinical features**: Isolated episode of	

© 2019 McCreary D et al. *JAMA Network Open.*

				LYST	c.5518T>G	p.Ser1840Ala	T/B/D	AR/Het	3	hemiplegia and ataxia. No systemic symptoms or other organ involvement. **Investigations:** MRI brain showed multiple white matter lesions in both hemispheres, posterior fossa, spinal cord with contrast enhancement and optic nerve swelling. Negative autoantibody profile and no acute phase response. **Treatment:** Nil	Unclassified neuroinflammatory disease
				FBN2	c.68C>G	p.Ala23Gly	D/B/N	AD/Het	3		
				ELN	c.259T>C	p.Phr87Leu	D/B/N	AD/Het	3		
				PEX2	c.748T>C	p.Trp250Arg	T/D/D	AR/Het	3		
				SPTAN1	c.965A>C	p. Gln322Pro	T/P/D	AD/Het	3		
				CLEC16A	c.1024C>G	p.Leu342Val	T/D/D	-/Het	3		
				PLCG2	c.2393A>G	p.Asn798Ser	D/D/D	AD/Het	3		
				NF1	c.2342A>T	p.His781Leu	T/B/D	AD/Het	3		
				LONP1	c.2485G>A	p.Ala829Thr	T/B/N	AR/Het	3		
				CBS;CBSL	c.833T>C	p.Ile278Thr	D/P/A	AR/Het	4		
58		F	8	*PLOD1*	c.607C>A	p.Arg203Ser	D/B/D	AR/Het	3	**Clinical features**: Microcephaly, seizure disorder. No systemic symptoms or other organ involvement. **Investigations:** MRI brain showed leukodystrophy and intracerebral calcification. Negative autoantibody profile and no acute phase response.	Unclassified neuroinflammatory disease
				CFH	c.737G>C	p.Arg246Thr	T/D/D	AD;AR/Het	3		
				CFHR5	c.329T>C	p.Val110Ala	D/P/N	AD/Het	3		
				COQ8A	c.25A>G	p.Ile9Val	D/B/D	AR/Het	3		
				LYST	c.8497G>A	p.Ala2833Thr	D/B/N	AR/Het	3		
				GFM1	c.77A>G	p.Lys26Arg	T/B/D	AR/Het	3		
				TMEM173	c.1124G>T	p.Arg375Leu	D/D/D	AD/Het	3		

© 2019 McCreary D et al. *JAMA Network Open.*

				EIF2B2	c.982A>G	p.Ile328Val	T/B/D	AR/Het	3	**Treatment:** Corticosteroids	
				FBN1	c.4277G>C	p.Gly1426Ala	T/D/D	AD/Het	3		
				MEFV	c.442G>C	p.Glu148Gln	D/D/P	AD;AR/Het	3		
				PSTPIP1	c.1115C>T	p.Ala372Val	D/D/D	AD/Het	3		
				TSC2	c.2348C>G	p.Thr783Ser	T/B/N	AD/Het	3		
59		M	1	*CFHR5*	c.1067G>A	p.Arg356His	T/P/N	AD/Het	3	**Clinical features**: Seizure disorder. No systemic symptoms or other organ involvement. **Investigations**: Intracerebral calcification on CT brain. Negative autoantibody profile and no acute phase response. **Treatment:** Nil	Unclassified neuroinflammatory disease
				CFHR5	c.1412G>A	p.Gly471Glu	D/P/N	AD/Het	3		
				TGFBR2	c.374del	p.Lys128Serfs*34	./././.	AD/Het	3		
				NOTCH1	c.4372G>A	p.Ala1458Thr	D/B/N	AD/Het	3		
				MVK	c.877G>A	p. Val293Met	T/P/N	AD;AR/Het	3		
				PEX12	c.785_787del	p.Asp262_Trp263delinsG	./././.	AR/Het	3		
				RNF213	c.11882A>G	p.Glu3961Gly	T/B/N	-/Het	3		
				PRX	c.731C>T	p.Ala244Val	T/B/N	AD;AR/Het	3		
60		M	10	*YY1AP1*	c.320G>A	p.Gly107Glu	D/B/D	AR/Het	3	**Clinical features**: Left sided hemiplegia and progressive motor function deterioration, ataxia.	Unclassified neuroinflammatory disease
				CFHR5	c.1067G>A	p.Arg356His	T/P/N	AD/Het	3		
				LYST	c.8806G>A	p.Val2936Ile	T/B/D	AR/Het	3		

© 2019 McCreary D et al. *JAMA Network Open.*

				IFIH1	c.2138C>G	p.Thr713Ser	T/B/D	AD/Het	3	**Investigations**: MRI brain showed diffuse predominantly white matter diseases in right frontal lobe involving the ispilateral deep grey matter and extending into the right cerebral peduncle. Brain biopsy suggestive of perivascular inflammatory process predominantly lymphocytic. Negative autoantibody profile. **Treatment:** corticosteroids, cyclophosphamide and mycophenolate mofetil.	
				TSC1	c.2194C>T	p.His732Tyr	D/P/A	AD/Het	3		
				NOTCH1	c.4129C>T	p.Pro1377Ser	T/B/N	AD/Het	3		
				COL4A1	c.161C>T	p.Pro54Leu	D/D/D	AD/Het	3		
				RNF213	c.5114C>A	p.Thr1705Lys	D/./D	-/Het	3		
				RNF213	c.9952A>G	p.Ile3318Val	T/B/N	-/Het	3		
				PRX	c.1483G>C	p.Glu495Gln	T/B/D	AD;AR/Het	3		
61		F	6	*LYST*	c.5518T>G	p.Ser1840Ala	T/B/D	AR/Het	3	**Clinical features**: Bilateral optic atrophy with severe visual loss, oral ulceration, arthralgia, fatigue. No systemic symptoms or other organ involvement **Investigations**: Negative autoantibody profile. Normal complement studies. **Treatment:** corticosteroids.	Unclassified neuroinflammatory disease
				RANBP2	c.7172C>G	p.Thr2391Ser	T/B/N	AD/Het	3		
				COL3A1	c.1165A>T	p.Asn389Tyr	D/B/N	AD/Het	3		
				GFM1	c.490C>T	p.Arg164Cys	D/D/D	AR/Het	3		
				C6	c.2087A>G	p.Asp696Gly	T/B/D	-/Het	3		
				C2	c.39_849del	p.Met280Asnfs*3	./././	AR/Het	3		
				PSMB8	c.689A>G	p.Tyr230Cys	D/B/D	AR/Het	3		

© 2019 McCreary D et al. *JAMA Network Open.*

				POLG	c.158_159insGCA	p.Gln55_Pro56insGln	./././.	AD;AR/Het	3		
				MYH11	c.5275G>A	p.Val1759Ile	T/B/N	AD/Het	3		
				RNF213	c.2122C>G	p.His708Asp	T/B/N	-/Het	3		
				RNF213	c.14194A>G	p.Lys4732Glu	T/B/N	-/Het	3		
				USP18	c.974A>G	p.Asn325Ser	T/B/N	AR/Het	3		
62		F	14	*LMNA*	c.1672G>A	p.Gly558Arg	D/P/D	AD;AR/Het	3	**Clinical features**: Retinitis. No systemic symptoms or other organ involvement. **Investigations**: MRI brain showed leucoencephalopathy. Negative autoantibody profile and no acute phase response. **Treatment:** Nil	Unclassified neuroinflammatory disease
				CYP27A1	c.1151C>T	p.Pro384Leu	D/D/D	AR/Het	3		
				AARS2	c.967G>A	p.Val323Met	D/D/D	AR/Het	3		
				POLG	c.156_158del	p. Gln55del	./././.	AD;AR/Hom	3		
				MEFV	c.910G>A	p. Gly 304Arg	D/B/N	AD;AR/Het	3		
				TRAP1	c.109C>T	p.Arg37Trp	T/B/N	- /Het	3		
63		F	15	*RANBP2*	c.6680A>G	p.Tyr2227Cys	D/D/D	AD/Het	3	**Clinical features**: Relapsing-remitting MS like disorder characterised by seizures associated with autistic spectrum disorder and significant developmental delay. **Investigations**: MRI brain showed multiple, supratentorial white	Multiple sclerosis
				SUMF1	c.95C>T	p.Ala32Val	T/P/N	AR/Het	3		
				PSMB8	c.208A>T	p.Thr70Ser	D/D/D	AR/Het	3		
				ITPR3	c.3489G>C	p.Glu1163Asp	T/B/D	AR/Het	3		
				PEX7	c.695G>A	p.Arg232Gln	D/P/D/	AR/Het	3		

© 2019 McCreary D et al. *JAMA Network Open.*

				ELN	c.1831G>A	p.Gly611 Arg	T/B/D	AD/Het	3	matter lesions mostly in periventricular/callosal location with other foci of abnormality in the deep and subcortical white matter. Negative autoantibody profile. Normal biotinidase, negative autoantibodies including Aq4/MOG. Positive OGB **Treatment:** corticosteroids, rituximab, IVIG	
				COL4A 1	c.1673C>T	p.Ala 558Val	./. /.	AD/Het	3		
				POLG	c.158_159ins GCA	p. Glyln55_ Pro56ins Gln	./. /.	AD;AR/Het	3		
				TRAP1	c.1432_1433 insCCG	Ser477_G ly478insA la	T/B/N	AR/Het	3		
				RNF213	c.9952A>G	p.Ile3318 Val	T/P/D	AD/Het	3		
				LPIN2	c.1159A>G	p.Lys387 Glu	T/P/D	-/Het	3		
				ERCC2	c.1426G>A	p.Val476I le	D/B/D	AR/Het	3		
				USP18	c.280G>A	p.Glu94L ys	D/D/N	AR/Het	3		
64		F	18m onths	*MASP2*	c.467G>A	p.Cys156 Tyr	D/D/D	AR/Het	3	**Clinical features:** Sensorineural hearing loss, cardiomyopathy, hepatosplenomegaly, squint, hypotonia. **Investigations**: MRI brain showed posterior fossa haemorhage and marked atrophy, distortion of cerebellum and abnormal brainstem. Periventicular calcification. Negative autoantibody profile and no systemic symptoms or other organ involvement.	Unclassified neuroinflamma tory disease
				C8A	c.1331G>A	p.Arg444 His	D/D/D	AR/Het	3		
				DGUO K	c.4G>T	p.Ala2Ser	T/B/N	AR/Het	3		
				SLC25A 12	c.870G>C	p.Glu290 Asp	T/B/D	AR/Het	3		
				GATA2	c.481C>G	p.Pro161 Ala	T/D/D	AD/Het	3		
				CLCN2	c.704G>A	p.Arg235 Gln	D/D/D	AD;AR/Het	3		
				C6	c.650T>C	p.Ile217T hr	T/B/N	AD/Het	3		
				CLEC16 A	c.2707G>A	p. Ala 903Thr	D/B/N	-/Het	3		

© 2019 McCreary D et al. *JAMA Network Open.*

				GFAP	c.1288C>T	p.Arg430Cys	D/P/D	AD/Het	3	**Treatment:** Corticosteroids	
				PRX	c.1490C?T	p.Pro497Leu	D/B/D	AD;AR/Het	3		
				PRX	c.1483G>C	p.Glu495Gln	T/B/D	AD;AR/Het	3		
				CBS;CBSL	c.833T>C	p.Ile278Thr	D/D/A	AR/Het	4		
				USP18	c.974A>G	p.Asn325Ser	T/B/N	AR/Het	3		
65		M	15	*PEX10*	c.820A>G	p.Thr274Ala	T/D/D	AR/Het	3	**Clinical features**: Recurrent fevers, developmental regression, and family history of lupus. **Investigations**: MRI brain showed white matter disease. Negative autoantibodies and no acute phase response. **Treatment:** Corticosteroids	Unclassified neuroinflammatory disease
				MASP2	c.1142C>T	p.Thr381Ile	D/D/D	AR/Het	3		
				C8A	c.1723C>T	p.Pro575Ser	T/B/N	AR/Het	3		
				RANBP2	c.6362A>G	p.Gln2121Arg	T/D/D	AD/Het	3		
				IFIH1	c.2767A>G	p.Ile923Val	T/D/D	AD/Het	3		
				GBE1	c.2017G>A	p.Ala673Thr	T/B/N	AR/Het	3		
				WDR1	c.743A>G	p.His248Arg	T/B/D	-/Het	3		
				NOTCH1	c.3836G>A	p.Arg1279His	T/B/D	AD/Het	3		
				NOTCH1	c.2542G>A	p.Glu848Lys	T/D/D	AD/Het	3		
				TSC2	c.1100G>A	p.Arg367Gln	T/D/D	AD/Het	3		
				MEFV	c.554G>T	p.Gly185Val	T/P/N	AD;AR/Het	3		
				MEFV	c.537C>G	p.Ser179Arg	D/P/N	AD;AR/Het	3		

© 2019 McCreary D et al. *JAMA Network Open.*

				TRAP1	c.1324G>A	p.Glu442 Lys	T/B/D	-/Het	3		
				CTC1	c.2162G>C	p.Gly 721 Ala	T/B/N	AR/Het	3		
				CTC1	c.1241G>C	p.Gly414 Ala	T/D/D	AR/Het	3		
				USP18	c.295G>A	p.Val99Il e	T/D/N	AR/Het	3		
				USP18	c.974A>G	p.Asn325 Ser	T/B/N	AR/Het	3		
66		M	10	*COL3A 1*	c.4165G>T	p.Ala138 9Ser	T/B/N	AD/Het	3	**Clinical features**: Common variable immunodeficiency, PANDAS like syndrome, colitis. **Investigations**: MRI brain non-specific white matter disease. Negative autoantibody profile. **Treatment:** IVIG	Unclassified neuroinflamma tory disease
				DDX58	c.212G>A	p.Arg71H is	D/D/N	AD/Het	3		
				CBL	c.2588A>G	p.Asn863 Ser	T/B/N	AD;Smu/Het	3		
				NOD2	c.3017dupC	p.Leu100 7Profs*1	./././.	AD;Mu/Hom	3		
				POLG2	c.1247G>C	p.Gly416 Ala	T/D/D	AD/Het	3		
				LPIN2	c.1654C>G	p.Pro552 Ala	D/D/D	-/Het	3		
				PRX	c.1483G>C	p.Glu495 Gln	T/B/N	AD;AR/Het	3		
67		M	15	*RANBP 2*	c.6680A>G	p.Tyr222 7Cys	D/D/D	AD/Hom	3	**Clinical features**: Common variable immunodeficiency, **Investigations:** PANDAS like syndrome, colitis. MRI brain non-specific white matter disease. Negative autoantibody profile. **Treatment:** IVIG	Unclassified – neuroinflamma tory disease
				FBN2	c.4312G>A	p.Glu143 8Lys	T/P/D	AD/Het	3		
				ITPR3	c.4366G>A	p.Glu145 6Lys	T/P/D	AR/Het	3		
				ITPR3	c.7301C>T	p.Ser2434 Leu	T/B/N	AR/Het	3		
				DDX58	c.212G>A	p.Arg71H is	D/D/D	AD/Het	3		

© 2019 McCreary D et al. *JAMA Network Open.*

				C5	c.3047C>T	p.Ala1016Val	T/D/D	AD/Het	3		
				CBL	c.2588A>G	p.Asn863Ser	T/B/N	AD/Het	3		
				NOD2	c.866A>G	p.Asn289Ser	T/B/D	AD/Het	3		
				POLG2	c.1247G>C	p.Gly416Ala	T/D/D	AD/Het	3		
				LPIN2	c.1654C>G	p.Pro552Ala	D/D/D	-/Het	3		
68		F	18	*PEX10*	c.820A>G	p.Thr274Ala	T/D/D	AR/Het	3	**Clinical features**: Thyroiditis, leucoencephalopathy, peripheral neuropathy, thrombocytopaenia. **Investigations:** MRI brain non progressive white matter disease. Thyroid antibodies positive, other autoantibodies negative **Treatment:** corticosteroids.	Unclassified neuroinflammatory disease
				GJC2	c.355G>C	p.Gly119Arg	T/P/N	AD;AR/Het	3		
				GATA2	c.439C>T	p.Pro147Ser	D/P/D	AD/Het	3		
				GUCY1A3	c.720C>G	p.Phe240Leu	T/B/N	AR/Het	3		
				OTULIN	c.439C>T	p.Pro147Ser	T/B/N	AR/Het	3		
				HSD17B4	c.1192T>C	p.Ser398Pro	T/B/N	AR/Het	3		
				LMNB1	c.872C>T	p.Ala291Val	D/B/D	AD/Het	3		
				PEX6	c.235G>C	p.Ala79Pro	T/B/N	AD;AR/Het	3		
				NOTCH1	c.4129C>T	p.Pro1377Ser	T/B/N	AD/Het	3		
				ERCC6	c.3965G>T	p.Gly1322Val	D/D/.	AD;AR/Het	3		
				STAT2	c.2473G>T	p. Gly 825C	T/B/N	AR/Het	3		
				TSC2	c.3100G>A	p.Val1034Ile	D/D/D	AD/Het	3		

© 2019 McCreary D et al. *JAMA Network Open.*

				MEFV	c.688G>A	p.E230K	D/B/N	AD;AR/Het	3		
				PLCG2	c.1258G>A	p.Ala420 Thr	T/B/N	AD/Het	3		
				NOTCH 3	c.4036C>T	p.His1346 Tyr	T/D/.	AD/Het	3		
69		M	8	*MFN2*	c.1574A>G	p.Asn525 Ser	T/B/N	AD;AR/Het	3	**Clinical features**: Autism and epilepsy. No systemic symptoms or other organ involvement **Investigations:** MRI brain showed non progressive white matter disease. Negative autoantibody profile. **Treatment:** Nil	Unclassified neuroinflammatory disease
				C1QC	c.8T>C	p.Val3Ala	T/B/N	AR/Het	3		
				C8A	c.1558C>T	p.Arg520 Cys	D/D/N	AR/Het	3		
				RANBP 2	c.1258A>G	p.Thr420 Ala	T/B/N	AD/Het	3		
				RANBP 2	c.2306G>A	p.Arg769 Gln	T/B/N	AD/Het	3		
				FBN2	c.3476T>C	p.Ile1159 Thr	D/P/D	AD/Het	3		
				RHOD	c.244C>T	p.Arg82Trp	D/D/D	-/Het	3		
				ATP7B	c.406A>G	p.Arg136 Gly	T/B/N	AR/Het	3		
				TSC2	c.5017G>C	p.Val1673Leu	D/B/N	AD/Het	3		
				NOD2	c.1621C>T	p.Arg541 Trp	D/D/N	AD;Mu/Het	3		
				DNASE 2	c.664G>A	p.Gly222 Arg	D/D/D	-/Het	3		
				NOTCH 3	c.5779G>A	p.Ala1927Thr	T/B/D	AD/Het	3		
				TYMP	c.194G>A	p.Ser65Asn	T/B/N	AR/Het	3		
				MEFV	c.442G>C	p.Glu148 Gln	D/D/P	AD;AR/Het	3		
70		F	6	*RANBP 2*	c.5941T>A	p.Ser1981 Thr	T/B/N	AD/Het	3	**Clinical features**: Arthralgia and	

© 2019 McCreary D et al. *JAMA Network Open.*

				DNASE 1L3	c.244G>C	p. Gly 82Arg	T/B/N	AR/Het	3	erythematous skin rash, headaches and cognitive decline.<br>**Investigations:** MRI brain showed white matter disease with T2 progressive enhancing lesions.<br>No acute phase response, MPO antibodies positive on one occasion, negative autoantibody screen since.<br>Sibling of patient 66.<br>**Treatment:** corticosteroids	Unclassified neuroinflammatory disease
				SAMD3	c.1112C>T	p.Thr371Ile	T/P/D	-/Het	3		
				ELN	c.1043G>A	p.Gly348Glu	D/D/D	AD/Het	3		
				STAT2	c.2473G>T	p.Gly825Cys	T/B/N	AR/Het	3		
				POLG	c.158_159insGCA	p.Gln55_Pro56insGln	./././.	AD/Het	3		
				HSD17B4	c.1438-2A>G	p.Intronic	././D	AR/Het	3		
71		M	18	*RANBP2*	c.3162G>T	p. Gln1054His	T/B/D	AD/Het	3	**Clinical features**: Peripheral neuropathy and erythematous skin rashes.<br>**Investigations:** MRI brain showed non progressive white matter disease.<br>GAD antibodies positive, remaining autoantibody screen negative.<br>**Treatment:** corticosteroids	Unclassified neuroinflammatory disease
				RANBP2	c.3163C>T	p.Pro1055Ser	T/B/D	AD/Het	3		
				CLCN2	c.2103G>C	p.Glu701Asp	T/B/N	AD;AR/Het	3		
				CLCN2	c.2012G>A	p.Arg671Gln	T/B/D	AD;AR/Het	3		
				CLCN2	c.1883_1884insCCG	p.Arg629_Gln630insArg	./././.	AD;AR/Hom	3		
				ERCC8	c.1012G>A	p. Asp338Asn	T/B/D	AR/Het	3		
				TGFBI	c.1061A>G	p.Asn354Ser	D/P/D	AD/Het	3		
				SAMD3	c.1112C>T	p.T371Ile	T/P/D	-/Het	3		
				ELN	c.1043G>A	p. Gly 348Glu	D/D/D	AD/Het	3		

© 2019 McCreary D et al. *JAMA Network Open.*

				PEX2	c.748T>C	p.Trp250 Arg	T/D/D	AR/Het	3		
				TBK1	c.1391T>C	p.Val464 Ala	T/B/D	AD/Het	3		
				POLG	c.158_159ins GCA	p.Gln55_ Pro56ins Gln	./ ./.	AD;AR/Het	3		
				POLG	c.G125A	p.Arg42G ln	./ ./.	AD;AR/Het	3		
				NOTCH 3	c.3399C>A	p.His1133 Gln	T/B/N	AD/Het	3		
				PRX	c.4077_4079 del	p.Glu136 1del	./ ./.	AD;AR/Het	3		
				HSD17 B4	c.1438-2A>G	p.Introni c	./ ./D	AR/Het	3		
72		M	3	*C1QC*	c.559G>A	p. Val 187Ile	T/B/N	AR/Het	3	**Clinical features**: Episode of HSV encephalitis in infancy treated with antivirals; recurrence of seizures age 4 years old. No other systemic symptoms or other organ involvement **Investigations:** MRI brain showed widespread asymmetrical cortical and subcortical thalamic enhancing lesions on MRI brain. Brain biopsy revealed granulomatous inflammation. Negative autoantibodies, no acute phase response. **Treatment:** corticosteroids.	Chronic HSV granulomatous encephalitis
				RANBP 2	c.7172C>G	p.Thr239 1Ser	T/B/N	AD/Het	3		
				TGFBR 2	c.449del	p.Lys153 Serfs*34	./ ./.	AD/Het	3		
				PEX1	c.3283G>A	p.Asp109 5Asn	T/D/D	AR/Het	3		
				POLG2	c.1105A>G	p.Arg369 Gly	D/D/D	AD/Het	3		
				ERCC2	c.442C>T	p.His148 Tyr	T/B/D	AR/Het	3		
				NLRP12	c.3088C>G	p.Arg103 0Gly	D/D/N	AD/Het	3		

© 2019 McCreary D et al. *JAMA Network Open.*

73		F	15	*SDHB*	c.487T>C	p.Ser163Pro	T/B/A	AD/Het	3	**Clinical features**: Papilloedema and headaches, raised ICP, arthralgia but no other systemic symptoms **Investigations:** MRI brain showed white matter insular and frontal cortex lesions. No acute phase response, autoantibodies negative. **Treatment:** corticosteroids.	Unclassified neuroinflammatory disease
				TRNT1	c.1232T>C	p.Ile411Thr	D/P/D	AR/Het	3		
				C5	c.3029C>T	c.Thr1232Cys	T/D/D	AD/Het	3		
				NOTCH1	c.3836G>A	p.Arg1279His	T/B/D	-/Het	3		
				DNASE1	c.619C>T	p.Arg207Cys	D/D/D	AD/Het	3		
				ACOX1	c.1830A>T	p.Glu610Asp	T/B/D	AR/Het	3		
				RNF213	c.14030G>T	p.Trp4677 Leu	D/D/D	-/Het	3		
				PRX	c.4077_4079del	p.Glu1361del	./././	AD;AR/Het	3		
74		F	4	*MASP2*	c.881C>T	p.Thr294Met	D/B/N	AR/Het	3	**Clinical features**: Raised ICP, right sided sixth nerve palsy and ophthalmological examination revealed papilloedema. **Investigations:** MRI brain showed bilateral subdural haemorrhages and left sided cortical swelling with involvement of the caudate nucleus and hippocampus and bilateral shallow subdural collections, left being more acute than the right of uncertain aetiology, possibly an acute on	Unclassified neuroinflammatory disease
				TGFBR2	c.1234G>A	p.Val412Met	T/D/D	AD/Het	3		
				HFE	c.829G>A	p.Glu277lys	D/D/D	AR/Het	3		
				PEX1	c.1380A>C	p.Glu460Asp	T/B/D	-/Het	3		
				COL4A1	c.1673C>T	p.Ala558Val	T/B/D	AD/Het	3		
				IRF8	c.982T>G	p.Phe328Val	D/B/N	AD;AR/Het	3		
				CTC1	c.671G>A	p.Arg224Gln	T/B/N	AR/Het	3		

© 2019 McCreary D et al. *JAMA Network Open.*

										chronic process. Repeat scans showed increased ventricular dilatation and persistent filling defect in the right sigmoid sinus thought to represent a non-occlusive thrombus. CT Angiogram - No radiological features to suggest a mid or large vessel vasculitis.<br>No acute phase response, autoantibodies negative, CSF raised neopterins (105nmol/l, RR<65 nmol/l), raised blood lactate at first presentation (7.9 and 4.3), CSF lactate normal, no infection identified.<br>**Treatment:** dexamethasone and underwent a burr-hole procedure and evacuation of sub-dural haemorrhage.	
75		M	16	*MASP2*	c.881C>T	p.Thr294Met	D/B/N	AR/Het	3	**Clinical features**: Developmental delay, dystonia. No systemic symptoms or other organ involvement	Unclassified neuroinflammatory disease
				PLOD1	c.376T>G	p.Phe126Val	D/D/D	AR/Hom	3		
				C8A	c.176G>A	p.Arg59Gln	D/D/D	AR/Het	3		

				C8A	c.1742C>G	p.Thr581 Arg	T/P/D	AR/Het	3	**Investigations:** Bilateral striatal necrosis on MRI brain imaging. Negative autoantibody screen. Negative CSF OGB. **Treatment:** Nil	
				PTPN22	c.550A>G	p.Thr184 Ala	T/B/D	AD;AR/Het	3		
				RANBP 2	c.1730A>G	p.Ala550 Gly	D/B/N	AD/Het	3		
				IFIH1	c.2431A>G	p.Thr811 Ala	D/D/D	AD/Het	3		
				SCN9A	c.1838C>T	p.Pro613 Leu	D/B/D	AD;AR/Het	3		
				SAMD3	c.569T>C	p.Leu190 Pro	D/D/D	-/Het	3		
				SAMD3	c.241C>G	p.Leu81V al	T/B/N	-/Het	3		
				ELN	c.328G>A	p.Ala110 Thr	D/D/D	AD/Het	3		
				NDUFA F1	c.536G>A	p.Arg179 Gln	T/B/N	AR/Het	3		
				CTC1	c.139A>G	p.Lys47G lu	T/D/N	AR/Het	3		
				ATPAF2	c.310T>C	p.Tyr104 His	T/B/D	AR/Het	3		
				RNF213	c.15017C>T	p.Ser5006 Leu	T/B/B	-/Het	3		
				LPIN2	c.2621G>T	p.Cys874 Phe	T/B/D	-/Het	3		
				MEFV	c.442G>C	p.Glu148 Gln	D/D/P	AD;AR/Het	3		
				CFHR5	c.970+2T>G	-	./../D	AD/Het	3		
76		M	7	*CFHR5*	c.1067G>A	p.Arg356 His	T/P/N	AD/Het	3	**Clinical features**: Recurrent fevers, headaches, arthralgia, loss of memory, seizures.	Unclassified neuroinflamma tory disease
				RANBP 2	c.7172C>G	p.Thr239 1Ser	T/B/N	AD/Het	3		

© 2019 McCreary D et al. *JAMA Network Open.*

				BTD	c.133G>A	p. Gly 45Arg	T/B/N	AR/Het	3	**Investigations:** MRI brain non progressive white matter disease, normal ESR and CRP, normal SAA, negative autoantibodies. **Treatment:** corticosteroids	
				TGFBR 2	c.449del	p.Lys153 Serfs*34	./././.	AD/Het	3		
				PEX6	c.235G>C	p.Ala79Pro	T/B/N	AD;AR/Het	3		
				POLG	c.158_159ins GCA	p.Gln55_ Pro56ins Gln	./././.	AD;AR/Het	3		
				PEX26	c.457C>G	p.Leu153 Val	T/B/N	AD;AR/Het	3		

© 2019 McCreary D et al. *JAMA Network Open.*

eFigure. Further Tests in Patients With Isolated Neuroinflammation

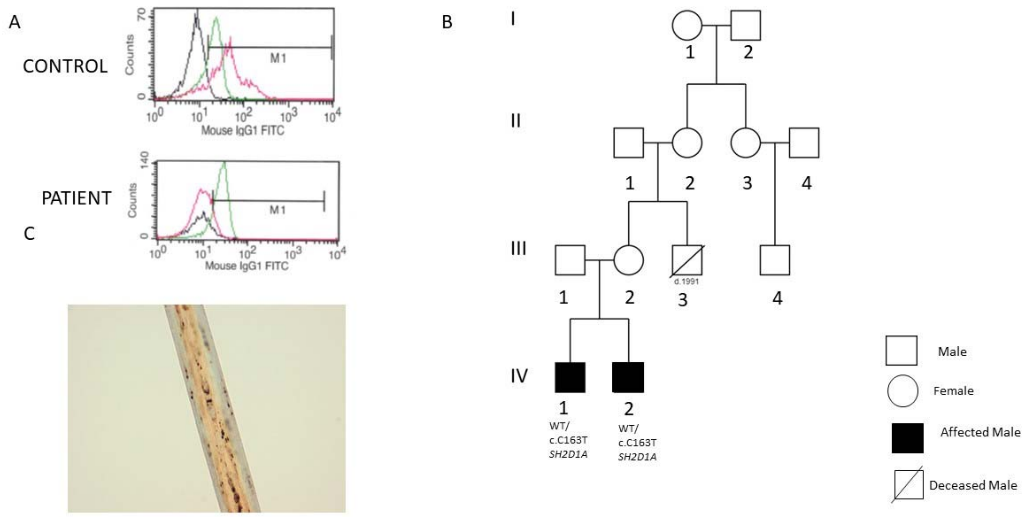

eFigure. (A) Absent signaling-lymphocytic-activation-molecule-associated protein (SAP) protein expression in lymphocytes derived from a 3 year old patient with isolated neuroinflammation due to X linked lymphoproliferative disease. (B) Pedigree for family of same patient (IV-1) diagnosed with X linked lymphoproliferative disease due to heterozygous NM_002351:c.C163T (p.Arg55Ter) in the SH2 Domain Containing 1A (*SH2D1A*) gene. His sibling (IV-2) also carried the same mutation. (C) Hair shaft examination from patient 42, showing prominent, diffuse clumps of melanin pigment, typical of Griscelli syndrome. She was identified on NIP to have two variants NM_004580:c.C550T (p.Arg184Ter) and NM_004580:c.G259C (p.Ala87Pro) variants in the Member RAS Oncogene Family *(RAB27A*) gene.

© 2019 McCreary D et al. *JAMA Network Open.*
